# The Alterations in Mitochondrial DNA Copy Number and Nuclear-Encoded Mitochondrial Genes in Rat Brain Structures after Cocaine Self-Administration

**DOI:** 10.1007/s12035-016-0153-3

**Published:** 2016-11-07

**Authors:** Anna Sadakierska-Chudy, Agnieszka Kotarska, Małgorzata Frankowska, Joanna Jastrzębska, Karolina Wydra, Joanna Miszkiel, Edmund Przegaliński, Małgorzata Filip

**Affiliations:** 1 Laboratory of Drug Addiction Pharmacology, Institute of Pharmacology Polish Academy of Science, Smetna 12, 31-343, Krakow, Poland; 2 Faculty of Biology and Earth Science, Neurobiology, Jagiellonian University, Krakow, Poland

**Keywords:** Cocaine self-administration, Gene expression, Microarray, mtDNA copy number, Mitochondrial dynamics, Oxidative stress

## Abstract

The repeated intake of cocaine evokes oxidative stress that is present even during drug withdrawal. Recent studies demonstrate that cocaine-induced oxidative and/or endoplasmic reticulum stress can affect mitochondrial function and dynamics as well as the expression of mitochondrial and nuclear genes. These alterations in mitochondrial function may determine synaptic and behavioral plasticity. Mitochondria and mitochondrial DNA (mtDNA) seem to play an important role in the initiation of drug addiction. We used a microarray approach to investigate the expression patterns of nuclear-encoded genes relevant for mitochondrial functions and quantitative real-time PCR assays to determine the numbers of copies of mtDNA and of mRNAs corresponding to two mitochondrial proteins in the prefrontal cortex and hippocampus of rats during early cocaine abstinence. We found a significant elevation in the copy number of mtDNA and concomitant increased expression of mitochondrial genes. Moreover, microarray analysis revealed changes in the transcription of nuclear genes engaged in mtDNA replication, nucleoid formation, the oxidative phosphorylation pathway, and mitochondrial fission and fusion. Finally, we observed the upregulation of endoplasmic reticulum stress-induced genes. Cocaine self-administration influences the expression of both nuclear and mitochondrial genes as well as mtDNA replication. To determine whether these alterations serve as compensatory mechanisms to help maintain normal level of ATP production, further studies are necessary.

**Electronic supplementary material:**

The online version of this article (doi:10.1007/s12035-016-0153-3) contains supplementary material, which is available to authorized users.

## Introduction

Cocaine is commonly abused illicit drug that stimulates brain reward circuits by increasing extraneuronal dopamine levels. The neurotoxic mechanism of cocaine may involve a number of factors including toxicity from dopamine metabolism and/or reactive oxygen species (ROS) formation as well as mitochondrial dysfunctions [1]. Cocaine exposure impairs mitochondrial functions through the inhibition of respiratory chain complex I and the reduction of the mitochondrial membrane potential [2, 3]. Mitochondria are crucial for neuronal projection and synaptic plasticity; they maintain optimal synaptic neurotransmission by generating adequate amounts of ATP and buffering Ca^2+^ [4, 5]. For example, hippocampal mitochondria are involved in the morphogenesis and plasticity of spines and synapses [6, 7].

A growing body of evidence indicates an association between mitochondrial DNA (mtDNA) and neuron structure as well as one between mtDNA and axonal and synaptic activity [8]. The mitochondrial genome contains only 37 genes, 13 of which encode proteins of four oxidative phosphorylation (OXPHOS) complexes, but approximately 70 structural proteins and more than 100 other proteins required for the incorporation of cofactors are encoded by nuclear DNA (nDNA) [9, 10]. Thus, mitochondrial OXPHOS requires mutual cooperation between the nuclear and mitochondrial genomes.

Interestingly, several studies suggest that cocaine can affect the expression of mitochondrial and nuclear genes as well as mitochondrial dynamics through oxidative stress (OS). In addition to producing energy, mitochondria are both generators and direct targets of ROS [11, 12]. In particular, mainly complex I and III of the respiratory chain are involved in the formation of the free radical superoxide, which in turn induces cellular OS [13]. The significance of OS and its mechanism of action in the development of drug addiction have been investigated by several groups. As previously demonstrated, cocaine treatment induces oxidative damage in the hippocampus (HIP) of rats exposed in utero [14], elevates OS markers in rat prefrontal cortex (PFC) and nucleus accumbens [15], or increases ROS production in the frontal cortex and the striatum [16]. Interestingly, we recently showed that cocaine self-administration enhanced superoxide dismutase (SOD) activity in the HIP, frontal cortex, and dorsal striatum suggesting the local increase of superoxide radical [17]. These changes are long lasting and persisted for up to 10 days following the withdrawal of cocaine where not only the SOD activity but also the level of malondialdehyde (a lipid peroxidation marker) was significantly elevated in the rat HIP and frontal cortex [18]. Further supporting the significance of imbalance in redox homeostasis in cocaine behavioral neuroplasticity, Uys et al. observed decreased expression of GSH-S-transferase pi that catalyzes the *S*-glutathionylation of cysteine residues on cellular proteins, in the nucleus accumbens of rats withdrawn from daily cocaine [19]. A study by Lee and Wei revealed that OS may influence the abundance of mitochondria as well as the integrity and copy number of mtDNA [11]. Additionally, mitochondrial proteins encoded by nDNA are thought to be the major determinant of the increased mtDNA copy number in response to OS [11].

Neurons are sensitive to changes in mitochondrial dynamic properties (fission, fusion, and transport) that facilitate energy distribution throughout neuronal projections [18]. Interestingly, mitochondrial dynamics is controlled by mtDNA quality as well as extra- and intracellular signals including OS and mitochondrial membrane potential [20]. Mitochondria undergo continual cycles of fusion and fission that result in the intermixing of the mitochondrial populations in the cell. Fusion and fission events, which are crucial for maintaining mitochondrial function, are regulated by several nuclear-encoded proteins including mitofusin 1 (*Mfn1*) and *Mfn2*, *Opa1*, *Drp1*, and *Fis1* [21]. It is believed that mitochondrial fusion helps cells address the increased energy demand during stress conditions [22]. Fusion allows the exchange of contents between mitochondria and is an essential process for protecting the integrity and stability of mtDNA [23]. It has been established that redistribution of the electron transport chain during mitochondrial fusion enhances ROS production, resulting in the increased mtDNA copy number [24]. In turn, the increased mtDNA content provides enough copies of mtDNA for continual cycles of fusion and fission and prevents the generation of mitochondria without mtDNA [24, 25]. Berman et al. found that mitochondrial fission is required to create new mitochondria and that the rate of fission exceeds the rate of fusion in healthy neuronal processes [26]. Interestingly, neuronal stimulation (electrical or pharmacological) increases mitochondrial trafficking in axons [27], and the fusion/fission process generates shorter mitochondria that are more mobile. Indeed, transport and distribution of mitochondria are required for maintaining synaptic strength and plasticity [28]. Additionally, learning and memory can lead to upregulation of nuclear-encoded mitochondrial genes [29]. These findings suggest that alterations in mitochondrial function may determine behavioral plasticity.

Here, we have used microarray and real-time PCR analysis to characterize the expression patterns of nuclear-encoded genes relevant for mitochondrial functions as well as the copy number of mtDNA and the transcript levels of two mitochondrial genes encoding subunits of OXPHOS complex I in the brain structures of rats during early cocaine abstinence. We performed analyses in the PFC and HIP, brain regions that are involved in cocaine reward and drug-seeking behavior [30]. Overall, our results show a significant increase in the copy number of mtDNA that is accompanied by increased expression of mitochondrial genes. In addition, the microarray analysis has revealed changes in the expression of nuclear genes involved in mtDNA replication, nucleoid formation, the OXPHOS pathway, and mitochondrial fission and fusion.

## Results

### Behavioral Experiment

Animals self-administering (SA) cocaine showed stable lever-pressing rates during the last 3 SA days, with less than a 10 % difference in their daily intake of cocaine. During 12 experimental sessions, animals received an average of 100 mg/kg of cocaine. Rats in the SA group pressed significantly more frequently on the active than on the inactive lever from the 2nd till the 14th experimental session, as assessed by the lever × day session interaction (*F*_(14,168)_ = 3.76, *p* < 0.001). In the yoked saline (YS) group, the difference in pressing the active vs. the inactive lever failed to reach significance (lever × day session interaction: *F*_(14,168)_ = 0.26)*.*

During extinction training (3 days), when cocaine was replaced by saline, the active lever presses decreased till 36 % in the SA group compared to control.

### Nuclear-Encoded Mitochondrial Genes Affected by Cocaine Self-Administration

**Fig. 1:**
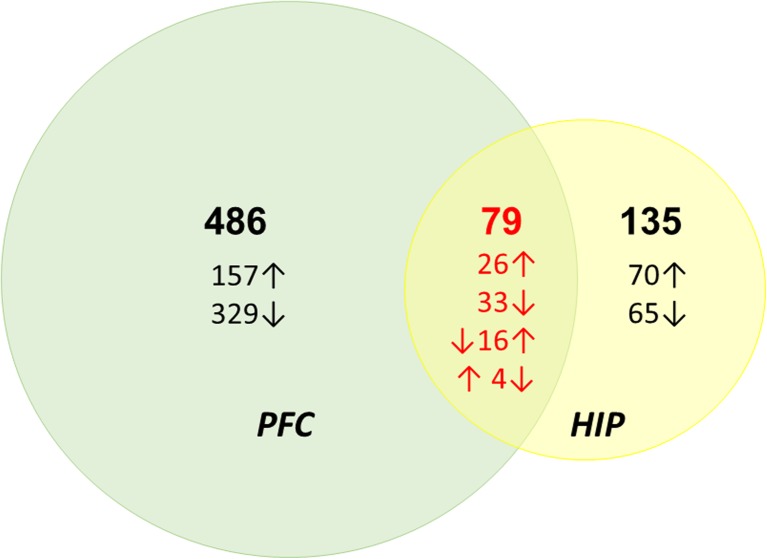
Differentially expressed nuclear genes encoding mitochondrial proteins in the brain structures of rats after cocaine self-administration compared to yoked saline controls. *PFC* prefrontal cortex, *HIP* hippocampus. Increased expression is indicated by ↑ and decreased expression by ↓

Microarray analysis was performed to assess the PFC and hippocampal transcriptomes at 3 days following cocaine SA in both groups of rats. The use of an FDR ≤0.1 identified 486 and 135 differentially expressed nuclear genes with mitochondrial localization during short cocaine abstinence with mitochondrial protein localization in the PFC and the HIP, respectively (Supplementary Table S1 and Table S2). We also observed an overlap of differentially expressed genes between the PFC and the HIP: a total of 79 genes showed expression changes in both brain structures (Supplementary Table S3). The numbers of up- and downregulated genes in both brain regions of cocaine SA rats compared to yoked control rats are summarized in Fig. 1.

To identify pathways in a set of nuclear genes, pathway enrichment analysis was performed. Three KEGG pathways, OXPHOS, tricarboxylic acid (TCA) cycle, and pyruvate metabolism, were significantly enriched (*p* < 0.001) in the SA group. Our results indicate that the expression level of nuclear genes encoding OXPHOS proteins is affected in both brain structures following cocaine SA (Supplementary Table S3). Using adjusted FDR ≤0.1 and log_2_ FC ≥0.2 and ≤−0.2 as a cutoff, we found 66 genes showing differential expression in the PFC following cocaine SA, comprising 23 upregulated and 43 downregulated genes (Supplementary Fig. S1 and Table S4). However, the majority of the selected genes (16 out of 19 transcripts) are upregulated in the HIP; 3 of them encoded subunits of mitochondrial respiratory complex I (*Ndufb5*, *Ndufb3*, and *Ndufa10*) and another 3 assembly factors of complex I (*Ndufaf7, Ndufaf6*, and *Ndufaf2*) (Supplementary Fig. S1 and Table S4).

To determine their mitochondrial localization as well as their biological processes and molecular functions, selected nuclear genes were subjected to GO analysis. Our approach showed that for the cellular component analysis, differentially expressed genes are mostly located at the mitochondrial inner membrane and the mitochondrial matrix in both rat brain regions following cocaine SA (Table 1). In addition, interestingly, several transcripts (11 in the PFC and 5 in the HIP) are related to the formation of mitochondrial nucleoids (Table 1).

**Table 1:** Summary of gene ontology (GO) analysis

GO term	PFC		HIP	
	Count	*p* value	Count	*p* value
Cellular component (CC)				
Mitochondrial inner membrane (GO: 0005743)	77	2.15E-74	24	2.65E-24
Mitochondrial outer membrane (GO: 0005741)	23	2.28E-19	7	6.74E-07
Mitochondrial matrix (GO: 0005759)	56	1.27E-55	21	1.33E-23
Mitochondrial nucleoid (GO: 0042645)	11	1.61E-13	5	6.86E-06
Molecular function (MF)				
Transporter activity (GO: 0005215)	33	2.44E-6	12	3.47E-4
Electron carrier activity (GO: 0009055)	12	1.88E-11	6	1.01E-7
DNA binding (GO: 0003677)	5	9.94E-1	4	4.28E-1
Biological Process (BP)				
Mitochondrial respiratory chain complex assembly (GO: 0033108)	6	3.34E-7	4	1.32E-6
Mitochondrial fusion (GO: 0008053)	6	6.24E-9	1	1.00E+00
Mitochondrial fission (GO: 0000266)	4	1.57E-4	2	2.82E-3
Mitochondrial genome maintenance (GO: 0000002)	5	2.25E-8	3	3.59E-6
Mitochondrial translation (GO: 0032543)	5	2.02E-7	1	1.00E+00

**Fig. 2:**
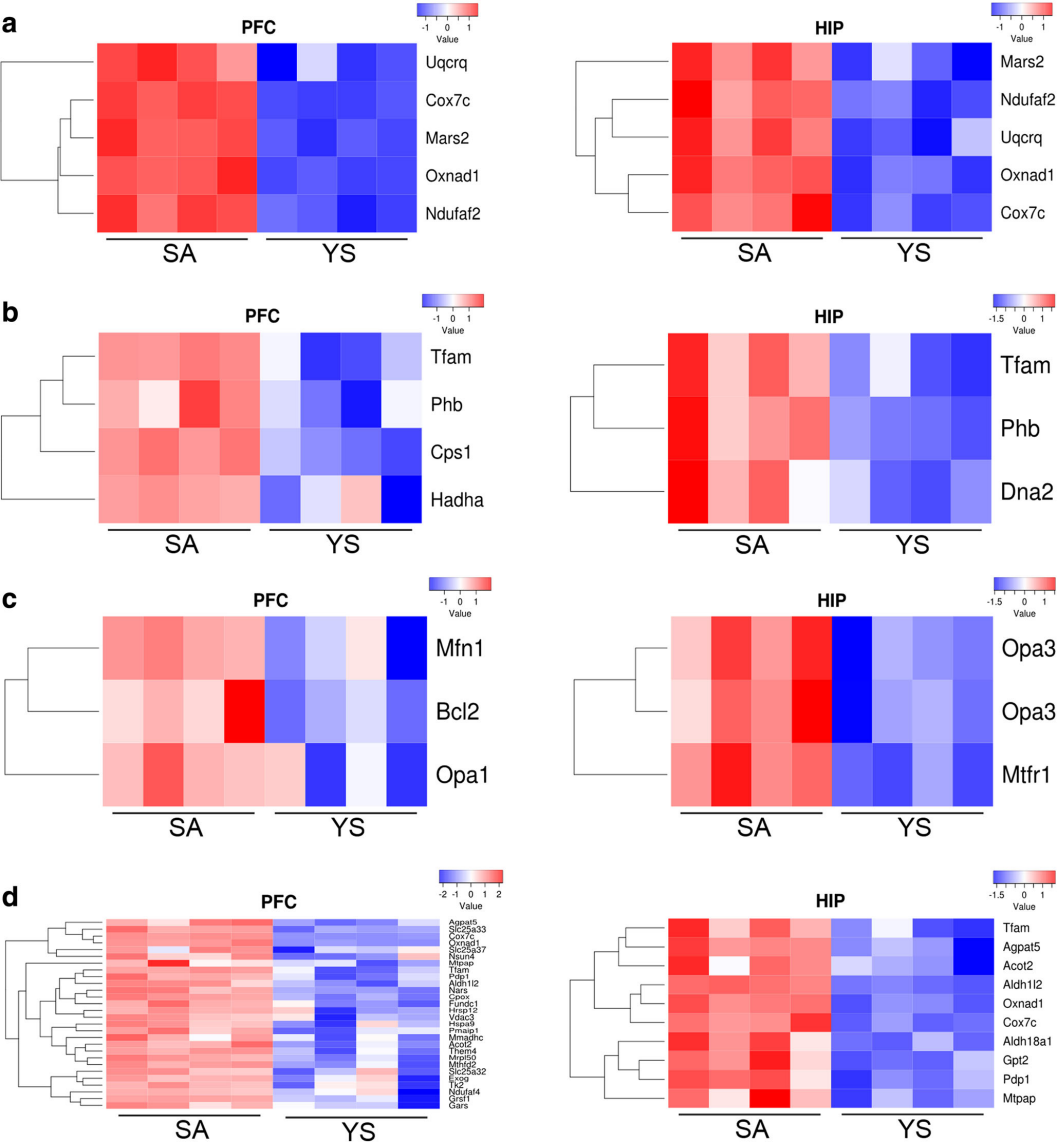
Gene expression changes in rat brain structures after cocaine self-administration. Microarray data are shown as heat maps displaying expression of the genes that were upregulated in the cocaine SA group vs. the YS group. The intensity of the color is proportional to the standardized values (between −2.5 to 2.5) from each microarray, as displayed on the bar below the heat map images. **a** The most upregulated genes. **b** Genes involved in mitochondrial nucleoid formation. **c** Genes involved in mitochondrial fusion and fission. **d** ER stress-response genes. *PFC* prefrontal cortex, *HIP* hippocampus, *SA* cocaine self-administration group, *YS* yoked saline group (control)

The highest upregulation across the two brain regions was observed for four genes (*Cox7c*, *Mars2*, *Ndufaf2*, *Oxnad1*, and *Uqcrq*) involved in energy generation and mitochondrial metabolism (Fig. 2a). Importantly, the transcript levels of three genes (*Tfam*, *Cps1*, and *Hadha*) in the PFC and three genes (*Tfam*, *Pbh*, and *Dna2*) in the HIP involved in nucleoid formation were moderately increased (Fig. 2b).

Quantifying the significance of GO biological processes, we identified genes involved in mitochondrial genome maintenance and mitochondrial fusion/fission (Supplementary Table S3). In the PFC, we identified several upregulated genes involved in mitochondrial dynamics: the fusion-related *Mfn1*, *Opa1*, and *Bcl2* and the fission-related *Mtfr1* and *Opa3* (Fig. 2c).

Recent data indicate that cocaine may induce ER stress; thus, we evaluated the expression of ER stress-induced genes encoding mitochondrial proteins. Using the cutoff values (see methods), we defined transcripts as upregulated in response to ER stress; there were a total of 28 and 10 ER stress-induced genes in the PFC and HIP, respectively (Fig. 2d). Interestingly, the upregulated genes are involved in energy metabolism (*Acot2*, *Cox7c*, and *Pdp1*), oxidoreductase activity (*Oxnad1*), and the maintenance and transcription of mtDNA (*Tfam* and *Mtpap*).

### mtDNA Copy Number

To verify that the DNA template yields result within the linear dynamic range of an assay, the relative standard curves were prepared. Assay reproducibility was examined through the correlation between Ct number and the logarithm concentration of DNA template. The linear relationship for each gene was noticed and the slopes did not significantly differ <−1. Moreover, the calculated Ct_*ND1*_/Ct_β-globin_ values (Mt/N) for each dilution (SD = 0.02) indicated that mitochondrial DNA and nuclear DNA were proceeded correctly.

**Fig. 3:**
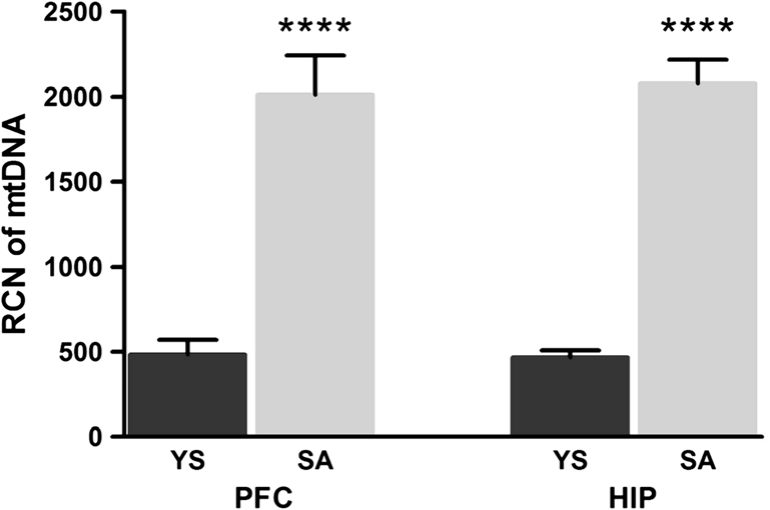
Relative mtDNA copy numbers in the rat prefrontal cortex and hippocampus following cocaine SA. *PFC* prefrontal cortex, *HIP* hippocampus, *RCN* relative copy number, *SA* cocaine self-administration group, *YS* yoked saline group (control) (****p ≤ 0.0001 vs. YS group; *N* = 7 animals/group; *error bars* = SEM)

To determine whether cocaine SA can initiate changes in the amount of mitochondrial genome persistence during extinction training, we quantified the relative copy number (RCN) of mitochondrial *ND1* vs. a nuclear gene in the PFC and HIP. We observed a statistically significant increase (by more than fourfold) in the number of mtDNA copies in each structure (Fig. 3).

### Levels of Mitochondrial Gene Expression

**Fig. 4:**
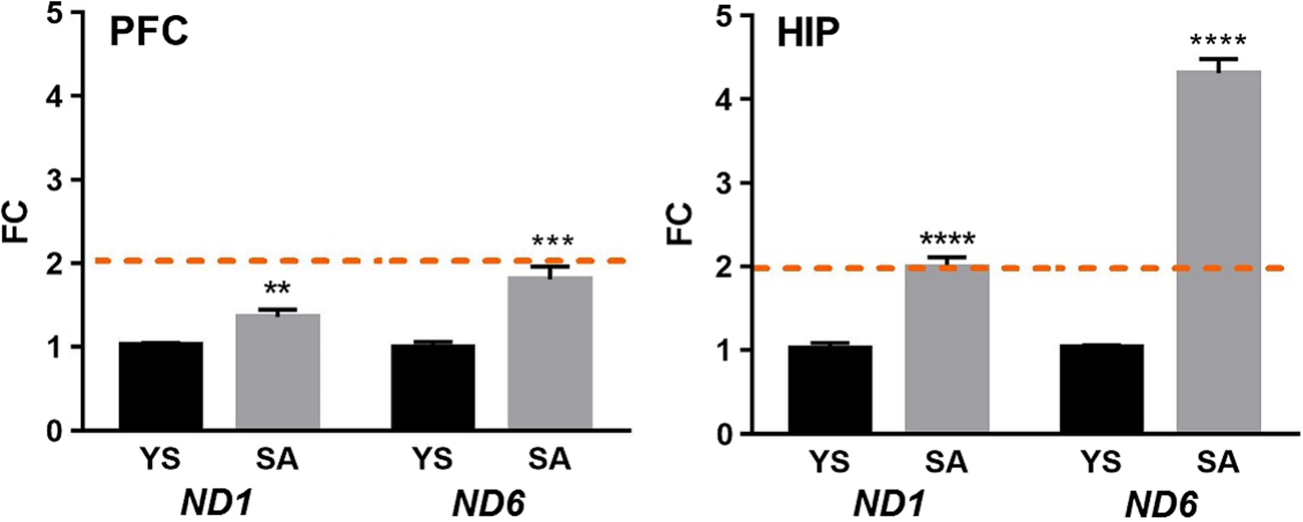
Mitochondrial gene expression in the brain structures following cocaine SA. *PFC* prefrontal cortex, *HIP* hippocampus, *FC* fold change (2^−ΔΔCt^), *SA* cocaine self-administration, *YS* yoked saline controls. (**p < 0.01, ***p < 0.001, ****p < 0.0001 vs. control; *N* = 6 animals/group; *error bars* = SEM; *dashed lines* indicate the twofold cutoff threshold value)

The expression of mitochondrial genes (*ND1* and *ND6*) encoding complex I subunits was assessed using real-time PCR. Gene expression was normalized to a housekeeping gene (*Hprt1*). There was no detection signal in the no-RT enzyme control (NEC) and the no-template control (NTC). In this study, the transcript levels of both mtDNA-encoded genes exceeded the fold change cutoff (>2) only in the HIP of SA rats (Fig. 4). We observed that mRNA levels of *ND1* and *ND6* in the HIP increased significantly, resulting in fold changes of 2.0 and 4.3, respectively (Fig. 4). In the PFC, the expression of *ND1* and *ND6* genes was higher (FC = 1.4 and 1.8, respectively) in the SA group than in the controls, but it did not reach the cutoff threshold.

## Discussion

In this study, we show cocaine-dependent significant differences in the mtDNA copy numbers in two rat brain structures engaged in addiction neuroadaptations. In fact, rats self-administering cocaine—in comparison to yoked saline controls—showed increases in mtDNA content of more than four times in the PFC and the HIP. Surprisingly, there is no data on the impact of cocaine on mtDNA copy number in rat brain during early (3-day) cocaine abstinence. A few studies have shown that addictive drugs decreased mtDNA copy number rather than increased. A recent study has demonstrated that chronic subcutaneous injection of morphine resulted in a decrease in the mtDNA copy number in the HIP and peripheral blood of addicted rats [7]. In contrast, our findings revealed that after cocaine self-administration, the amount of mtDNA drastically increased in both rat brain regions studied, with the concomitant elevation of the levels of two mitochondrial transcripts (*ND1* and *ND6*). These results may be explained in part by the fact that cocaine-evoked neuroplasticity (structural and synaptic plasticity) requires a large supply of energy.

According to our findings, cocaine SA can result in transcript-level changes in many important genes that are crucial for mitochondrial functions including energy production, nucleoid formation, and mitochondrial dynamics. We hypothesized that the observed increase in mtDNA copy number may help to maintain a normal level of mitochondrial transcripts to provide ATP. Indeed, our microarray data revealed changes in the expression levels of nuclear genes encoding proteins involved in oxidative phosphorylation (OXPHOS). Interestingly, four genes involved in OXPHOS pathways (i.e., *Ndufaf2*, *Uqcrq, Oxnad1*, and *Cox7c*) were significantly upregulated (Fig. 2a) in both brain regions. Our data are in agreement with results from a recent study showing that the increased demand for energy in human skeletal muscle during endurance exercises upregulates the expression of these genes [31]. Ndufaf2 is a complex I assembly factor that may act as a chaperone to assist in proper folding of proteins within respiratory chain complex I, thereby contributing to the proton gradient used for ATP generation [32, 33]. Furthermore, a recent report has highlighted the contribution of Ndufaf2 in OS and DNA damage: deficiency in this gene increased mitochondrial ROS and mtDNA deletions in a human knockdown neuroblastoma cell line as well as in mouse knockout fibroblasts [33]. Another upregulated gene, *Uqcrq* (encoding ubiquinol-cytochrome C reductase, complex III subunit VII), is essential for complex III activity [34]; moreover, the structural dependence between complex III and I has been confirmed [35]. Zhang et al. reported that self-administration of oxycodone (an opioid drug) increased the mRNA levels not only of *Uqcrq* but also of other genes encoding respiratory chain enzymes in the hypothalamus of adolescent mouse [36]. Further, *Oxnad1* (oxidoreductase NAD-binding domain containing 1) is involved in mitochondrial metabolism and is an ER stress-induced gene [37]. Importantly, a study by Pavlovsky et al. revealed that acute and repeated i.p. injection of cocaine (20 mg/kg and 2 × 20 mg/kg for 9 days, respectively) induced ER stress-response genes in the striatum, medial PFC, and nucleus accumbens [38]. In our study, in addition to those genes mentioned earlier, many nuclear OXPHOS transcripts were affected in either the PFC or the HIP (Supplementary Fig. 1S and Table 4S). Similarly, Lehrmann et al. reported that cocaine increased the levels of transcripts that participate in mitochondrial energy metabolism in the PFC of human cocaine addicts [39].

Moreover, our study shows that genes encoding proteins involved in mitochondrial nucleoid formation (including *Tfam*, *Dna2*, *Pbh*, *Hadha*, and *Cps1*) are also affected in the PFC and/or HIP (Fig. 2b). The main protein component of the nucleoid is a mitochondrial transcription factor (Tfam) that packs and organizes mtDNA. Tfam is necessary to maintain the integrity of mtDNA and is a key regulator of mitochondrial transcription and replication [40]. As previously demonstrated, disruption of the *Tfam* gene in mouse led to the loss of mtDNA [41–43], while its forced overexpression in HEK293 cell lines also resulted in mtDNA depletion [44]. Furthermore, Ekstrand et al. reported that mtDNA copy number in mice increased proportionally with mild increases in Tfam levels [42]. Likewise, we found that modest upregulation of the *Tfam* transcript was accompanied by increased mtDNA copy number in both the PFC and HIP. Thus, our results tend to confirm previous observations that even small changes in Tfam levels can induce changes in the copy number of mtDNA.

Several recent studies underscore the importance of mitochondrial dynamics—fusion, fission, and motility—in synapse density and plasticity, which are essential in the development of addiction [5, 45, 46]. Using gene expression microarray analysis, we found increased mRNA levels of genes (*Mfn1*, *Opa1*, *Mtfr1*, and *Opa3*) involved in mitochondrial fusion/fission in the PFC in SA rats (Fig. 2c). Interestingly, the importance of mitochondrial fusion was previously shown using a double knockout of the genes *Mfn1* and *Mfn2* in mouse embryonic fibroblasts (MEFs) [47]. Chen and coworkers revealed that the lack of *Mfn1* and *Mfn2* completely abolishes fusion and causes severe cellular defects including the reduction of the mtDNA copy number and problems with ATP synthesis [47]. It is well known that Opa1 (optic atrophy factor 1) mediates inner mitochondrial membrane fusion and requires Mfn1 to promote this process [48, 49]. Furthermore, observations in *S. cerevisiae* and MEFs indicate that Opa1 and MFN proteins are essential for maintaining the integrity of mtDNA nucleoids [50] as well as for mtDNA replication and distribution [51]. It is believed that fusion allows the optimization of mitochondrial function in cases of increased energy demand during stress conditions [22]. A recent study has elegantly demonstrated that mitochondrial fusion generates ROS, which in turn induces mtDNA replication, thereby preventing the formation of mitochondria lacking mtDNA nucleoids [24]. Furthermore, we also observed differentially expressed genes involved in the regulation of mitochondrial fission—mitochondrial fission regulator 1 (*Mtfr1*) and optic atrophy 3 (*Opa3*). An in vitro study revealed that Mtfr1 induces the mitochondrial transition from network to spheroid architecture [52], while in vitro analysis performed on the testis mitochondria-enriched fraction showed that *Mtfr1* is also involved in the regulation of antioxidant activity and mitochondrial respiration as well as ATP synthesis [53]. In addition, a previous study reported that Opa3, an integral protein of the mitochondrial outer membrane, affects mitochondrial fission and probably interacts with Mfn1 [54]. Altogether, it is very likely that mitochondrial fusion and fission help to maintain mitochondrial DNA integrity following cocaine-induced OS.

It is well established that endoplasmic reticulum (ER) is in physical contact with the mitochondria and that stress from either the ER or mitochondria affects the other. Recently, Go et al. demonstrated that both acute and repeated cocaine injections induced ER stress in rat dorsal striatum [55]. Similarly, we observed upregulation of ER stress-responsive genes such as *Oxnad1*, *Cox7c*, and *Tfam* (Fig. 2d). Moreover, many studies have demonstrated that cocaine enhanced mitochondrial ROS production through its metabolites, leading to cellular OS in several brain structures implicated in the circuitry of addiction, including the frontal cortex, striatum, nucleus accumbens, and HIP [15, 56–59].

Assessing the significance of previous findings, we assumed that the increases in mtDNA copy number and transcript levels of nuclear genes that were observed in our experiments may result from OS evoked by excessive ROS generation and/or ER stress induced by cocaine itself or its metabolites and/or from the increased concentration of dopamine in the synaptic cleft. Our data may be partly supported by the results of a recent study showing that the relative mtDNA copy number significantly increased due to OS in the frontal cortex of autistic subjects [60]. Additionally, Lee et al. showed that OS after H_2_O_2_ treatment increased the mtDNA copy number in human lung fibroblasts [61]. Based on these observations, we hypothesized that higher mtDNA copy number may be a compensatory mechanism to increase the number of wild-type templates for maintaining a normal level of ATP production.

We have to note that there are some limitations of the present study. First, the changes in the level of transcripts in brain structures were not confirmed at the protein level. Next, we did not simultaneously assess the activity of respiratory chain complexes to determine whether cocaine SA can influence energy production. Thus, these issues will be considered in our future studies.

## Conclusions

In this study, we observed that mtDNA copy number increased concomitantly with mitochondrial gene expression in the PFC and the HIP in rats after cocaine SA. The current study identified differentially expressed nuclear genes involved in the OXPHOS pathway, and GO enrichment analysis identified genes mediated in nucleoid formation, DNA replication, and mitochondrial fusion and fission. We postulate that cocaine may increase the copy number of mtDNA and alterations in the expression of mitochondrial and nuclear genes through increased ROS production leading to OS and/or ER stress. A key aim in the future will be to assess the levels of oxidative mtDNA damage, respiratory chain enzymatic activity, and mitochondrial dynamics in both of these structures in the rat brain.

## Methods

### Behavioral Procedures

#### Animals

Male Wistar rats (290–350 g; Charles River Laboratories, Germany) were housed 5 per cage (during initial training) or individually (during the rest of the procedures) in standard plastic rodent cages, with free access to food (Labofeed pellets), in a colony room maintained at 40 ± 5 % humidity, 20 ± 1 °C, and under a 12-h light–dark cycle (lights on at 6:00 a.m.). Water was provided ad libitum during the whole experiment except for the initial self-administration training and retraining following the surgery, during which it was provided 2 h/day after sessions. All procedures were conducted during the light phase of the light–dark cycle (between 8:00 a.m. and 3:00 p.m.).

#### Operant Conditioning—Initial Training

After 7 days of initial self-administration, training was performed in standard operant conditioning cages under a fixed ratio (FR) schedule of water reinstatement. Briefly, rats were deprived of water (18 h) before the training and were rewarded with water (0.1 ml) in operant chambers following 1 press on the “active” lever (FR1), which increased to 3 (FR3), and finally to 5 (FR5) lever presses. An arbitrary operant behavior (lever-pressing) acquisition criterion required that the animal achieved 100 reinforcements during a single session under the FR1, FR3, and FR5 schedule.

#### Surgical Procedures

Animals anesthetized by intramuscular injection of saline solution of ketamine hydrochloride (75 mg/kg; Bioketan, Biowet, Poland) and xylazine hydrochloride (5 mg/kg; Sedazin, Biowet, Poland) underwent silastic catheter implantation into an external jugular vein [62]. The catheters were flushed daily with 0.2 ml of cephazolin (10 mg/ml, Tarfazolin, Polfa, Poland) dissolved in heparinized saline solution (70 U/ml, Polfa, Poland).

#### Self-Administration and Extinction Training

During 12 2-h daily sessions, the animals were given free access to the drug—pressing on the active lever (FR5) resulted in a 5-s infusion of 0.1 ml of cocaine (0.5 mg/kg, Sigma-Aldrich, USA) paired with the conditioning stimuli (cue light and tone, 2000 Hz, 15 dB above ambient noise level). Each infusion was followed by a 20-s time-out when rats could not receive the drug despite pressing the lever. An arbitrary operant behavior (lever-pressing) acquisition criterion required that subjects’ active lever presses vary by 10 % over 3 consecutive maintenance days.

After the self-administration sessions, the animals underwent 3-day extinction training, during which the same operant chamber was used for 2-h sessions, but cocaine was replaced with saline (0.1 ml/infusion) and no conditioning stimuli were presented. On the last day of extinction training, the animals were sacrificed immediately after the 2-h session. Brain structures (PFC and HIP) were dissected out and rapidly frozen using dry ice then stored at −80 °C.

#### Yoked Self-Administration Procedure

The yoked procedure allows the pharmacological and motivational effects of a psychostimulant to be distinguished. In this procedure, rats actively self-administering cocaine (SA) were paired with rats passively receiving saline (YS—yoked saline, control group). Unlike SA rats, lever pressing by the YS rats had no programmed consequences.

### Molecular Procedures

#### Extraction of Nucleic Acids

The isolation of DNA and RNA was performed using the RNA/DNA/PROTEIN Purification Plus Kit (Norgen Biotek, Canada) with a minor modification to the manufacturer’s protocol. Briefly, the frozen brain structures (10–15 mg) were homogenized using the Bioprep-24 Homogenizer (Aosheng, China) (30 s at 3000 rpm, then 2 × 30 s at 2500 rpm) in the presence of ceramic beads (*ø* = 2.8 mm) and 350 μl of lysis buffer. Then, the sample was transferred to a new tube and incubated in a thermoblock (ThermoMixer, Eppendorf) at 30 °C for 3 min, followed by shaking at 500 rpm for 2 min. Finally, precipitation and wash steps were performed to purify nucleic acids. DNA samples were eluted in nuclease-free water (Sigma-Aldrich, USA) and stored at −20 °C until further use. RNA samples were eluted in nuclease-free water preheated to 60 °C, followed by removal of the trace of DNA by treatment with DNase I (Qiagen, USA) using the RNA Clean-Up kit (Syngen, Poland) according to the manufacturer’s recommendation, and the purified RNA was eluted in nuclease-free water and stored at −80 °C until further analysis.

The quantity and quality of the isolated DNA and RNA were determined using a NanoDrop ND-1000 Spectrophotometer (Thermo Scientific, USA) and agarose gel electrophoresis. DNA samples with low 260/230 ratios were further purified using the gDNA Clean-Up Kit (Syngen, Poland) following the manufacturer’s protocol. Additionally, RNA integrity was evaluated using a Bioanalyzer (Agilent Technologies, USA).

#### Microarray Analysis

The Rat 4x44K Gene Expression Array v2 (Agilent Technologies, USA), representing 39,000+ rat genes and transcripts, was used to assess gene expression in rat brain structures. Sample labeling and hybridization were performed according to the Agilent One-Color Microarray-Based Gene Expression Analysis protocol. Briefly, eight RNA samples at equal concentration were pooled into 4 sets in each of the two groups (SA and YS), and then 100–200 μg of total RNA was amplified and labeled with Cy3-UTP. The concentration and specific activity of cRNAs were determined using a NanoDrop ND-100 (Thermo Scientific, USA). The labeled cRNAs (1 μg) were fragmented and hybridized to the array for 17 h at 65 °C with rotation. Two post-hybridization washes were performed to remove nonspecific hybridization, and the array was fixed and scanned. Image acquisition and feature extraction for the array were performed using the Agilent Microarray Scanner and Feature Extraction software (v 11.0.1.1) (Agilent Technologies, USA). Subsequent quantile normalization and data processing were carried out using the GeneSpring GX software, v. 12.1 (Agilent Technologies, USA). For the analysis, we used genes that were significantly different in expression (log_2_ FC ≥0.2 and ≤−0.2, FDR ≤0.1) between the SA and YS groups. To select differentially expressed nuclear-encoded and mitochondrial genes, the Mouse MitoCarta2.0 dataset (www.broad.mit.edu/publications/MitoCarta) was used. To identify genes and pathways associated with specific mitochondrial functions, Gene Ontology (GO) and KEGG pathway enrichment analyses were performed using the STRING database v10.0 (http://string-db.org/) to obtain the enriched cellular components, biological processes, and pathways. *p* < 0.001 was set as the threshold value.

#### Real-Time PCR: mtDNA Copy Number and Gene Expression

To quantify mtDNA copy number, two genes representing either mitochondrial or nuclear DNA were used (*ND1* and β-globin genes, assay ID Rn03296764_s1 and Rn04223896_s1, respectively). Total genomic DNA (3 μg) was mixed with 5 μl of TaqMan Expression Master Mix (Life Technologies, USA), 0.5 μl of TaqMan assay (Life Technologies, USA), and nuclease-free water to adjust the final volume to 10 μl. To avoid dilution bias, a serial dilution of the DNA template (with five concentrations from 10 to 1.25 ng) for both genes was prepared in duplicate.

To assess the expression of mitochondrial genes, total RNA (700 ng) and random hexamer primers were used for reverse transcription reactions. The cDNAs were synthesized in a total volume of 20 μl with the Transcription High Fidelity cDNA Synthesis Kit (Roche) following the manufacturer’s protocol. Then, PCR reaction mixtures (10 μl) containing 4.5 μl of cDNA (diluted 1:1 in nuclease-free water), 5 μl of TaqMan Expression Master Mix (Life Technologies, USA), and 0.5 μl of TaqMan assay for *ND1* (Rn03296764_s1) or *ND6* (Rn03296815_s1) or *Hprt1* (Rn01527840_m1) (Life Technologies, USA) were prepared. All real-time PCRs were performed in duplicate in a 96-well plate and run in a BioRad CFX96 Touch™ Real-Time PCR Detection System. The following thermal conditions were used: 95 °C for 10 min, followed by 40 cycles of 15 s at 95 °C and 60 s at 60 °C. NEC and NTC controls were included in all RT reactions.

The threshold cycle (Ct) was collected using CFX Manager*™* software. The copy number of the mitochondrial gene *ND1* was normalized to a single-copy nuclear gene, β-globin. Relative mtDNA copy number (RCN) was calculated by a comparative Ct method, using the following equation: RCN = 2^ΔCt^, where ΔCt = Ct_β-globin_−Ct_*ND1*_. Relative quantification for mitochondrial gene expression was performed using the ∆∆Ct method. The expression of target genes was normalized to *Hprt1*, and YS samples were used for calibration. Fold changes of >2.0 or <0.5 were used as cutoff thresholds (*p* < 0.05) to identify up̶̶̶̶̶regulated or downregulated genes, respectively.

#### Statistical Analysis

The obtained results are presented as the means ± SEM. Behavioral data were analyzed in Statistica software (v. 10) using a two-way ANOVA for repeated measures followed by a post hoc Newman-Keuls test. The molecular data (mtDNA copy number and mitochondrial gene expression) were analyzed by Student’s *t* test using GraphPad Prism software (v. 5.04). R software (v. 3.1.2) was used to conduct Student’s *t* test followed by the Benjamini and Hochberg correction for microarray data. *p* values <0.05 were considered statistically significant.

## Electronic Supplementary Material

Supplementary Figure S1 (PNG 213 kb)

Supplementary Table S1 (DOCX 45 kb)

Supplementary Table S2 (DOCX 22 kb)

Supplementary Table S3 (DOCX 19 kb)

Supplementary Table S4 (DOCX 17 kb)
