# Supplementary figures and images for: The Alterations in Mitochondrial DNA Copy Number and Nuclear-Encoded Mitochondrial Genes in Rat Brain Structures after Cocaine Self-Administration
Source: Mol Neurobiol. 2016 Nov 7;54(9):7460–70. doi: 10.1007/s12035-016-0153-3 (PMC5622911; doi:10.1007/s12035-016-0153-3)

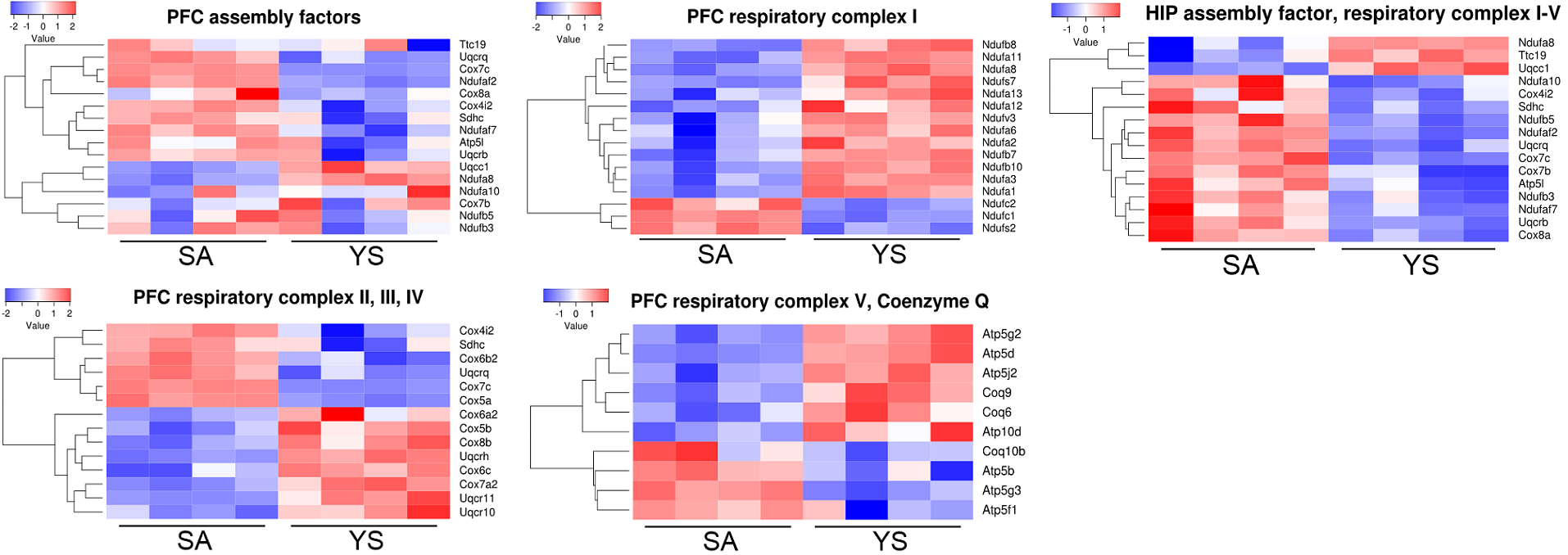

Supplement: Supplementary file 1 — (PNG 213 kb) [file 12035_2016_153_MOESM1_ESM.png]
