# Supplementary material for: The Alterations in Mitochondrial DNA Copy Number and Nuclear-Encoded Mitochondrial Genes in Rat Brain Structures after Cocaine Self-Administration
Source: Mol Neurobiol. 2016 Nov 7;54(9):7460–70. doi: 10.1007/s12035-016-0153-3 (PMC5622911; doi:10.1007/s12035-016-0153-3)
Supplement: Supplementary file 2 — (DOCX 45 kb) [file 12035_2016_153_MOESM2_ESM.docx]

**Table 1S. Nuclear genes encoding mitochondrial proteins with significant differential expression (FDR≤0.1; log_2_FC**≥**0.2 and ≤ -0.2) observed in rat prefrontal cortex during the 3^rd^ day of extinction training after cocaine self-administration.**

| **Gene symbol** | **Gene name** | **log_2_ FC** | ***P* value** |
| --- | --- | --- | --- |
| *Abcb6* | ATP-binding cassette, subfamily B (MDR/TAP), member 6 | -0.8 | 0.00 |
| *Abcb8* | ATP-binding cassette, subfamily B (MDR/TAP), member 8 | -0.4 | 0.03 |
| *Abcb9* | ATP-binding cassette, subfamily B (MDR/TAP), member 9 | -0.3 | 0.01 |
| *Abcd1* | ATP-binding cassette, subfamily D (ALD), member 1 | -0.4 | 0.05 |
| *Abcd2* | ATP-binding cassette, subfamily D (ALD), member 2 | 0.3 | 0.01 |
| *Abcd3* | ATP-binding cassette, subfamily D (ALD), member 3 | -0.3 | 0.00 |
| *Abhd11* | abhydrolase domain containing 11 | -0.3 | 0.01 |
| *Acaa1a* | acetyl-CoA acyltransferase 1 | -0.7 | 0.00 |
| *Acaa1b* | acetyl-Coenzyme A acyltransferase 1B | -0.6 | 0.00 |
| *Acaa2* | acetyl-CoA acyltransferase 2 | -0.7 | 0.01 |
| *Acad10* | acyl-CoA dehydrogenase family, member 10 | -0.7 | 0.00 |
| *Acad11* | acyl-CoA dehydrogenase family, member 11 | -0.5 | 0.02 |
| *Acad8* | acyl-CoA dehydrogenase family, member 8 | -0.3 | 0.01 |
| *Acad9* | acyl-CoA dehydrogenase family, member 9 | 0.2 | 0.01 |
| *Acadl* | acyl-CoA dehydrogenase, long chain | -0.2 | 0.00 |
| *Acadm* | acyl-CoA dehydrogenase, C-4 to C-12 straight chain | -0.2 | 0.02 |
| *Acads* | acyl-CoA dehydrogenase, C-2 to C-3 short chain | -0.5 | 0.00 |
| *Acadvl* | acyl-CoA dehydrogenase, very long chain | -0.7 | 0.00 |
| *Acat1* | acetyl-CoA acetyltransferase 1 | 0.4 | 0.00 |
| *Acn9* | ACN9 homolog (S. cerevisiae) | -0.3 | 0.00 |
| *Aco2* | aconitase 2, mitochondrial | -0.3 | 0.02 |
| *Acot2* | acyl-CoA thioesterase 2 | 0.6 | 0.00 |
| *Acp6* | acid phosphatase 6, lysophosphatidic | -0.3 | 0.03 |
| *Acsf2* | acyl-CoA synthetase family member 2 | 0.5 | 0.04 |
| *Acsf3* | acyl-CoA synthetase family member 3 | -0.3 | 0.01 |
| *Acsl6* | acyl-CoA synthetase long-chain family member 6 | 0.5 | 0.03 |
| *Acss1* | acyl-CoA synthetase short-chain family member 1 | -0.3 | 0.04 |
| *Acss3* | acyl-CoA synthetase short-chain family member 3 | -0.5 | 0.02 |
| *Acyp2* | acylphosphatase 2, muscle type | -0.3 | 0.00 |
| *Adck2* | aarF domain containing kinase 2 | -0.3 | 0.01 |
| *Adck3* | aarF domain containing kinase 3 | -0.4 | 0.00 |
| *Adck4* | aarF domain containing kinase 4 | -0.5 | 0.03 |
| *Adhfe1* | alcohol dehydrogenase, iron containing, 1 | -0.4 | 0.00 |
| *Afg3l1* | AFG3(ATPase family gene 3)-like 1 (S. cerevisiae) | -0.3 | 0.04 |
| *Afg3l2* | AFG3-like AAA ATPase 2 | -0.4 | 0.00 |
| *Agpat5* | 1-acylglycerol-3-phosphate O-acyltransferase 5 | 0.4 | 0.00 |
| *Agxt2* | alanine-glyoxylate aminotransferase 2 | -0.6 | 0.02 |
| *Aifm2* | apoptosis-inducing factor, mitochondrion-associated 2 | -0.5 | 0.00 |
| *Aifm3* | apoptosis-inducing factor, mitochondrion-associated 3 | -0.7 | 0.00 |
| *Ak2* | adenylate kinase 2 | -0.4 | 0.05 |
| *Akap10* | A kinase (PRKA) anchor protein 10 | 0.2 | 0.03 |
| *Akr1b7* | aldo-keto reductase family 1, member B7 | -1.2 | 0.03 |
| *Alas1* | aminolevulinate, delta-, synthase 1 | -0.4 | 0.00 |
| *Alas2* | aminolevulinate, delta-, synthase 2 | -1.1 | 0.03 |
| *Aldh1l1* | aldehyde dehydrogenase 1 family, member L1 | -0.5 | 0.00 |
| *Aldh1l2* | aldehyde dehydrogenase 1 family, member L2 | 0.4 | 0.00 |
| *Aldh2* | aldehyde dehydrogenase 2 family (mitochondrial) | -0.5 | 0.00 |
| *Aldh5a1* | aldehyde dehydrogenase 5 family, member A1 | -0.4 | 0.00 |
| *Aldh7a1* | aldehyde dehydrogenase 7 family, member A1 | -0.4 | 0.00 |
| *Amacr* | alpha-methylacyl-CoA racemase | -0.2 | 0.04 |
| *Apoa1bp* | apolipoprotein A-I binding protein | -0.2 | 0.01 |
| *Asah2* | N-acylsphingosine amidohydrolase (non-lysosomal ceramidase) 2 | 0.7 | 0.01 |
| *Atad3a* | ATPase family, AAA domain containing 3A | -0.4 | 0.00 |
| *Atp10d* | ATPase, class V, type 10D | -0.4 | 0.01 |
| *Atp5b* | ATP synthase, H+ transporting, mitochondrial F1 complex, beta polypeptide | 0.5 | 0.01 |
| *Atp5d* | ATP synthase, H+ transporting, mitochondrial F1 complex, delta subunit | -0.5 | 0.00 |
| *Atp5f1* | ATP synthase, H+ transporting, mitochondrial Fo complex, subunit B1 | 0.2 | 0.04 |
| *Atp5g2* | ATP synthase, H+ transporting, mitochondrial Fo complex, subunit C2 (subunit 9) | -0.2 | 0.00 |
| *Atp5g3* | ATP synthase, H+ transporting, mitochondrial Fo complex, subunit C3 (subunit 9) | 0.9 | 0.00 |
| *Atp5j* | ATP synthase, H+ transporting, mitochondrial Fo complex, subunit F6 | 0.2 | 0.00 |
| *Atp5j2* | ATP synthase, H+ transporting, mitochondrial Fo complex, subunit F2 | -0.3 | 0.00 |
| *Atp5l* | ATP synthase, H+ transporting, mitochondrial Fo complex, subunit G | -0.2 | 0.01 |
| *Atp5sl* | ATP5S-like | -0.2 | 0.05 |
| *Atpaf1* | ATP synthase mitochondrial F1 complex assembly factor 1 | 0.8 | 0.00 |
| *Auh* | AU RNA binding protein/enoyl-CoA hydratase | 0.4 | 0.01 |
| *Aurkaip1* | aurora kinase A interacting protein 1 | -0.3 | 0.01 |
| *Bad* | BCL2-associated agonist of cell death | -0.4 | 0.00 |
| *Bax* | Bcl2-associated X protein | -0.4 | 0.00 |
| *Bcat2* | branched chain amino acid transaminase 2, mitochondrial | -0.8 | 0.00 |
| *Bckdha* | branched chain ketoacid dehydrogenase E1, alpha polypeptide | -0.6 | 0.00 |
| *Bckdk* | branched chain ketoacid dehydrogenase kinase | -0.4 | 0.01 |
| *Bcl2* | B-cell CLL/lymphoma 2 | 2.8 | 0.02 |
| *Bcl2l13* | BCL2-like 13 (apoptosis facilitator) | 0.8 | 0.02 |
| *Bcl2l2* | Bcl2-like 2 | 0.2 | 0.00 |
| *Bcs1l* | BC1 (ubiquinol-cytochrome c reductase) synthesis-like | 0.2 | 0.02 |
| *Bdh1* | 3-hydroxybutyrate dehydrogenase, type 1 | 0.4 | 0.01 |
| *Bid* | BH3 interacting domain death agonist | 0.3 | 0.04 |
| *Bloc1s1* | biogenesis of lysosomal organelles complex-1, subunit 1 | -0.3 | 0.01 |
| *Bnip3l* | BCL2/adenovirus E1B interacting protein 3-like | -0.2 | 0.00 |
| *Bola1* | bolA family member 1 | -0.4 | 0.03 |
| *Bphl* | biphenyl hydrolase-like (serine hydrolase) | -0.2 | 0.02 |
| *Car5b* | carbonic anhydrase 5b, mitochondrial | 0.4 | 0.02 |
| *Carkd* | carbohydrate kinase domain containing | -0.2 | 0.04 |
| *Cbr4* | carbonyl reductase 4 | 0.6 | 0.00 |
| *Ccdc127* | coiled-coil domain containing 127 | 0.6 | 0.01 |
| *Ccdc58* | coiled-coil domain containing 58 | -0.3 | 0.00 |
| *Cct7* | chaperonin containing Tcp1, subunit 7 (eta) | -0.3 | 0.00 |
| *Cep89* | centrosomal protein 89kDa | -0.7 | 0.00 |
| *Chchd10* | coiled-coil-helix-coiled-coil-helix domain containing 10 | -0.2 | 0.01 |
| *Chchd5* | coiled-coil-helix-coiled-coil-helix domain containing 5 | -0.5 | 0.00 |
| *Chchd6* | coiled-coil-helix-coiled-coil-helix domain containing 6 | -0.3 | 0.01 |
| *Chdh* | choline dehydrogenase | -0.4 | 0.02 |
| *Cisd2* | CDGSH iron sulfur domain 2 | 0.4 | 0.01 |
| *Cisd3* | CDGSH iron sulfur domain 3 | -0.3 | 0.01 |
| *Cmpk2* | cytidine monophosphate (UMP-CMP) kinase 2, mitochondrial | -0.2 | 0.00 |
| *Coa3* | cytochrome C oxidase assembly factor 3 | 0.3 | 0.00 |
| *Coa4* | cytochrome c oxidase assembly factor 4 | -0.3 | 0.02 |
| *Coasy* | CoA synthase | -0.3 | 0.01 |
| *Comtd1* | catechol-O-methyltransferase domain containing 1 | -0.3 | 0.03 |
| *Coq10b* | coenzyme Q10 homolog B (S. cerevisiae) | 0.3 | 0.04 |
| *Coq6* | coenzyme Q6 monooxygenase | -0.4 | 0.01 |
| *Coq9* | coenzyme Q9 | -0.4 | 0.00 |
| *Cox11* | cytochrome c oxidase assembly homolog 11 (yeast) | 0.5 | 0.00 |
| *Cox14* | cytochrome c oxidase assembly protein 14 | -0.3 | 0.01 |
| *Cox16* | COX16 cytochrome c oxidase assembly homolog (S. cerevisiae) | 0.4 | 0.02 |
| *Cox17* | COX17 cytochrome c oxidase copper chaperone | -0.4 | 0.00 |
| *Cox18* | cytochrome c oxidase assembly protein 18 | -0.4 | 0.00 |
| *Cox19* | cytochrome c oxidase assembly homolog 19 (S. cerevisiae) | -0.2 | 0.00 |
| *Cox4i2* | cytochrome c oxidase subunit IV isoform 2 (lung) | 0.5 | 0.01 |
| *Cox5a* | cytochrome c oxidase, subunit Va | 0.4 | 0.00 |
| *Cox5b* | cytochrome c oxidase subunit Vb | -0.2 | 0.00 |
| *Cox6a2* | cytochrome c oxidase subunit VIa polypeptide 2 | -0.8 | 0.02 |
| *Cox6b2* | cytochrome c oxidase subunit VIb polypeptide 2 | 0.7 | 0.00 |
| *Cox6c* | cytochrome c oxidase, subunit VIc | -0.3 | 0.00 |
| *Cox7a2* | cytochrome c oxidase subunit VIIa polypeptide 2 | -0.4 | 0.00 |
| *Cox7c* | cytochrome c oxidase, subunit VIIc | 2.6 | 0.00 |
| *Cox8b* | cytochrome c oxidase, subunit VIIIb | -0.6 | 0.00 |
| *Cpox* | coproporphyrinogen oxidase | 0.8 | 0.00 |
| *Cps1* | carbamoyl-phosphate synthetase 1 | 0.6 | 0.00 |
| *Cpt1c* | carnitine palmitoyltransferase 1c | -0.5 | 0.00 |
| *Cpt2* | carnitine palmitoyltransferase 2 | -0.5 | 0.00 |
| *Cryz* | crystallin, zeta (quinone reductase) | 0.3 | 0.01 |
| *Cyb5b* | cytochrome b5 type B (outer mitochondrial membrane) | 0.4 | 0.02 |
| *Cyb5r1* | cytochrome b5 reductase 1 | 0.3 | 0.04 |
| *Cyp11b2* | cytochrome P450, family 11, subfamily b, polypeptide 2 | 2.1 | 0.00 |
| *Cyp27a1* | cytochrome P450, family 27, subfamily a, polypeptide 1 | -0.6 | 0.00 |
| *Dap3* | death associated protein 3 | -0.3 | 0.02 |
| *Dbi* | diazepam binding inhibitor (GABA receptor modulator, acyl-CoA binding protein) | -0.3 | 0.00 |
| *Dcakd* | dephospho-CoA kinase domain containing | -0.4 | 0.00 |
| *Dcxr* | dicarbonyl L-xylulose reductase | -0.7 | 0.00 |
| *Dhodh* | dihydroorotate dehydrogenase (quinone) | -0.2 | 0.02 |
| *Dhrs4* | dehydrogenase/reductase (SDR family) member 4 | -0.3 | 0.03 |
| *Dlat* | dihydrolipoamide S-acetyltransferase | 0.6 | 0.01 |
| *Dlst* | dihydrolipoamide S-succinyltransferase (E2 component of 2-oxo-glutarate complex) | -0.3 | 0.00 |
| *Dmpk* | dystrophia myotonica-protein kinase | -0.9 | 0.05 |
| *Dna2* | DNA replication helicase/nuclease 2 | -0.3 | 0.04 |
| *Dnajc4* | DnaJ (Hsp40) homolog, subfamily C, member 4 | -0.5 | 0.00 |
| *Dnlz* | DNL-type zinc finger | -0.5 | 0.00 |
| *Ech1* | enoyl CoA hydratase 1, peroxisomal | -0.4 | 0.00 |
| *Echdc2* | enoyl CoA hydratase domain containing 2 | -0.3 | 0.02 |
| *Eci2* | enoyl-CoA delta isomerase 2 | -0.3 | 0.01 |
| *Ecsit* | ECSIT signalling integrator | -0.4 | 0.00 |
| *Eefsec* | eukaryotic elongation factor, selenocysteine-tRNA-specific | -0.4 | 0.00 |
| *Ephx2* | epoxide hydrolase 2, cytoplasmic | -0.2 | 0.04 |
| *Etfb* | electron-transfer-flavoprotein, beta polypeptide | -0.3 | 0.01 |
| *Exog* | endo/exonuclease (5'-3'), endonuclease G-like | 0.9 | 0.04 |
| *Fahd1* | fumarylacetoacetate hydrolase domain containing 1 | -0.3 | 0.00 |
| *Fam136a* | family with sequence similarity 136, member A | 0.4 | 0.00 |
| *Fam162a* | family with sequence similarity 162, member A | -0.4 | 0.00 |
| *Fam210a* | family with sequence similarity 210, member A | 0.7 | 0.02 |
| *Fam210b* | family with sequence similarity 210, member B | 0.7 | 0.02 |
| *Fastk* | Fas-activated serine/threonine kinase | -0.5 | 0.00 |
| *Fastkd1* | FAST kinase domains 1 | -0.6 | 0.00 |
| *Fdx1l* | ferredoxin 1-like | -0.4 | 0.00 |
| *Fdxr* | ferredoxin reductase | -0.4 | 0.00 |
| *Fech* | ferrochelatase | 0.3 | 0.04 |
| *Fhit* | fragile histidine triad | -0.5 | 0.00 |
| *Fis1* | fission 1 (mitochondrial outer membrane) homolog (S. cerevisiae) | -0.3 | 0.00 |
| *Fkbp8* | FK506 binding protein 8 | -0.3 | 0.03 |
| *Fth1* | ferritin, heavy polypeptide 1 | -0.3 | 0.01 |
| *Fundc1* | FUN14 domain containing 1 | 0.6 | 0.00 |
| *Fxn* | frataxin | -0.4 | 0.00 |
| *Gadd45gip1* | growth arrest and DNA-damage-inducible, gamma interacting protein 1 | -0.5 | 0.00 |
| *Gapdh* | glyceraldehyde-3-phosphate dehydrogenase | -0.3 | 0.00 |
| *Gars* | growth arrest-specific 1 | 0.6 | 0.02 |
| *Gbas* | glioblastoma amplified sequence | 0.2 | 0.04 |
| *Gcdh* | glutaryl-CoA dehydrogenase | -0.5 | 0.01 |
| *Gdap1* | ganglioside-induced differentiation-associated-protein 1 | 1.1 | 0.01 |
| *Gfm1* | G elongation factor, mitochondrial 1 | 0.2 | 0.00 |
| *Gfm2* | G elongation factor, mitochondrial 2 | 0.3 | 0.03 |
| *Ghitm* | growth hormone inducible transmembrane protein | 0.4 | 0.04 |
| *Glrx2* | glutaredoxin 2 | 0.2 | 0.05 |
| *Glrx5* | glutaredoxin 5 | -0.3 | 0.01 |
| *Gls* | glutaminase | -0.3 | 0.02 |
| *Gls2* | glutaminase 2 (liver, mitochondrial) | -0.3 | 0.00 |
| *Glyctk* | glycerate kinase | -0.6 | 0.00 |
| *Gng5* | guanine nucleotide binding protein (G protein), gamma 5 | -0.4 | 0.00 |
| *Golph3* | golgi phosphoprotein 3 (coat-protein) | 0.2 | 0.03 |
| *Gpd2* | glycerol-3-phosphate dehydrogenase 2, mitochondrial | 0.3 | 0.03 |
| *Gpx4* | glutathione peroxidase 4 | -0.2 | 0.00 |
| *Grhpr* | glyoxylate reductase/hydroxypyruvate reductase | -0.5 | 0.00 |
| *Grsf1* | G-rich RNA sequence binding factor 1 | 0.7 | 0.01 |
| *Gsr* | glutathione reductase | 1.8 | 0.02 |
| *Gstz1* | glutathione S-transferase zeta 1 | -0.3 | 0.01 |
| *Gtpbp3* | GTP binding protein 3 | -0.4 | 0.00 |
| *Gtpbp6* | GTP binding protein 6 (putative) | -0.6 | 0.00 |
| *Guk1* | guanylate kinase 1 | -0.3 | 0.02 |
| *Hadha* | hydroxyacyl-CoA dehydrogenase/3-ketoacyl-CoA thiolase/enoyl-CoA hydratase (trifunctional protein), alpha subunit | 1.2 | 0.04 |
| *Hadhb* | hydroxyacyl-CoA dehydrogenase/3-ketoacyl-CoA thiolase/enoyl-CoA hydratase (trifunctional protein), beta subunit | 0.2 | 0.03 |
| *Hars2* | histidyl-tRNA synthetase 2, mitochondrial | -0.2 | 0.00 |
| *Hccs* | holocytochrome c synthase | 0.5 | 0.00 |
| *Hdhd3* | haloacid dehalogenase-like hydrolase domain containing 3 | -0.8 | 0.03 |
| *Hemk1* | HemK methyltransferase family member 1 | -0.2 | 0.04 |
| *Higd1a* | HIG1 hypoxia inducible domain family, member 1A | 0.3 | 0.03 |
| *Hint1* | histidine triad nucleotide binding protein 1 | 0.3 | 0.01 |
| *Hint2* | histidine triad nucleotide binding protein 2 | -0.3 | 0.00 |
| *Hint3* | histidine triad nucleotide binding protein 3 | 0.2 | 0.01 |
| *Hk1* | hexokinase 1 | 0.2 | 0.00 |
| *Hmbs* | hydroxymethylbilane synthase | -0.4 | 0.00 |
| *Hrsp12* | heat-responsive protein 12 | 0.3 | 0.02 |
| *Hsd17b10* | hydroxysteroid (17-beta) dehydrogenase 10 | -0.3 | 0.00 |
| *Hspa9* | heat shock protein 9 | 0.9 | 0.02 |
| *Htra2* | HtrA serine peptidase 2 | -0.3 | 0.01 |
| *Iba57* | IBA57, iron-sulfur cluster assembly homolog (S. cerevisiae) | -0.3 | 0.02 |
| *Idh2* | isocitrate dehydrogenase 2 (NADP+), mitochondrial | -0.3 | 0.01 |
| *Ifi27* | interferon, alpha-inducible protein 27 | 0.2 | 0.04 |
| *Isca2* | iron-sulfur cluster assembly 2 | -0.2 | 0.01 |
| *Ivd* | isovaleryl-CoA dehydrogenase | -0.2 | 0.03 |
| *Kars* | lysyl-tRNA synthetase | -0.2 | 0.01 |
| *Kif1b* | kinesin family member 1B | 0.8 | 0.01 |
| *Krt5* | keratin 5 | -0.9 | 0.00 |
| *L2hgdh* | L-2-hydroxyglutarate dehydrogenase | 0.6 | 0.05 |
| *Lace1* | lactation elevated 1 | -0.4 | 0.00 |
| *Lactb* | lactamase, beta | 1.3 | 0.00 |
| *Lamc1* | laminin, gamma 1 | 0.4 | 0.00 |
| *Lap3* | leucine aminopeptidase 3 | 0.7 | 0.00 |
| *Ldhal6b* | lactate dehydrogenase A-like 6B | -0.4 | 0.05 |
| *Letm1* | leucine zipper-EF-hand containing transmembrane protein 1 | 0.2 | 0.01 |
| *Letmd1* | LETM1 domain containing 1 | -0.2 | 0.00 |
| *Lipt2* | lipoyl(octanoyl) transferase 2 (putative) | -0.3 | 0.01 |
| *Lonp1* | lon peptidase 1, mitochondrial | -0.3 | 0.00 |
| *Lypla1* | lysophospholipase I | 0.5 | 0.00 |
| *Lyrm2* | LYR motif containing 2 | -0.3 | 0.02 |
| *Macrod1* | MACRO domain containing 1 | -0.5 | 0.01 |
| *Malsu1* | mitochondrial assembly of ribosomal large subunit 1 | 0.2 | 0.04 |
| *Mars2* | methionyl-tRNA synthetase 2, mitochondrial | 2.6 | 0.00 |
| *Mccc1* | methylcrotonoyl-CoA carboxylase 1 (alpha) | -0.4 | 0.00 |
| *Mcur1* | mitochondrial calcium uniporter regulator 1 | 0.3 | 0.04 |
| *Mdh1* | malate dehydrogenase 1, NAD (soluble) | 0.2 | 0.02 |
| *Mdh2* | malate dehydrogenase 2, NAD (mitochondrial) | 0.3 | 0.00 |
| *Me2* | malic enzyme 2, NAD(+)-dependent, mitochondrial | 1.6 | 0.01 |
| *Me3* | malic enzyme 3, NADP(+)-dependent, mitochondrial | -0.2 | 0.01 |
| *Mecr* | mitochondrial trans-2-enoyl-CoA reductase | -0.4 | 0.00 |
| *Mettl17* | methyltransferase like 17 | -0.5 | 0.01 |
| *Mfn1* | mitofusin 1 | 0.9 | 0.02 |
| *Mfn2* | mitofusin 2 | -0.3 | 0.00 |
| *Mgarp* | mitochondria-localized glutamic acid-rich protein | 1.1 | 0.00 |
| *Minos1* | mitochondrial inner membrane organizing system 1 | -0.3 | 0.00 |
| *Mmadhc* | methylmalonic aciduria (cobalamin deficiency) cblD type, with homocystinuria | 0.3 | 0.02 |
| *Mpc2* | mitochondrial pyruvate carrier 2 | -0.4 | 0.00 |
| *Mpv17l2* | MPV17 mitochondrial membrane protein-like 2 | -0.3 | 0.01 |
| *Mrpl10* | mitochondrial ribosomal protein L10 | 0.7 | 0.00 |
| *Mrpl14* | mitochondrial ribosomal protein L14 | -0.2 | 0.04 |
| *Mrpl17* | mitochondrial ribosomal protein L17 | 0.8 | 0.00 |
| *Mrpl2* | mitochondrial ribosomal protein L2 | -0.3 | 0.01 |
| *Mrpl20* | mitochondrial ribosomal protein L20 | -0.2 | 0.02 |
| *Mrpl21* | mitochondrial ribosomal protein L21 | 0.6 | 0.00 |
| *Mrpl22* | mitochondrial ribosomal protein L22 | -0.2 | 0.01 |
| *Mrpl28* | mitochondrial ribosomal protein L28 | -0.3 | 0.01 |
| *Mrpl3* | mitochondrial ribosomal protein L3 | 0.5 | 0.03 |
| *Mrpl30* | mitochondrial ribosomal protein L30 | 0.3 | 0.03 |
| *Mrpl37* | mitochondrial ribosomal protein L37 | -0.2 | 0.00 |
| *Mrpl38* | mitochondrial ribosomal protein L38 | -0.5 | 0.01 |
| *Mrpl4* | mitochondrial ribosomal protein L4 | 0.3 | 0.00 |
| *Mrpl44* | mitochondrial ribosomal protein L44 | -0.2 | 0.05 |
| *Mrpl45* | mitochondrial ribosomal protein L45 | -0.2 | 0.03 |
| *Mrpl47* | mitochondrial ribosomal protein L47 | -0.9 | 0.02 |
| *Mrpl50* | mitochondrial ribosomal protein L50 | -0.3 | 0.03 |
| *Mrpl50* | mitochondrial ribosomal protein L50 | 0.6 | 0.00 |
| *Mrpl53* | mitochondrial ribosomal protein L53 | -0.4 | 0.00 |
| *Mrpl54* | mitochondrial ribosomal protein L54 | -0.4 | 0.00 |
| *Mrps11* | mitochondrial ribosomal protein S11 | -0.4 | 0.00 |
| *Mrps15* | mitochondrial ribosomal protein S15 | 1.6 | 0.01 |
| *Mrps16* | mitochondrial ribosomal protein S16 | -0.4 | 0.01 |
| *Mrps18a* | mitochondrial ribosomal protein S18A | -0.3 | 0.00 |
| *Mrps18c* | mitochondrial ribosomal protein S18C | -0.3 | 0.00 |
| *Mrps21* | mitochondrial ribosomal protein S21 | -0.9 | 0.00 |
| *Mrps22* | mitochondrial ribosomal protein S22 | 0.3 | 0.04 |
| *Mrps24* | mitochondrial ribosomal protein S24 | -0.3 | 0.00 |
| *Mrps25* | mitochondrial ribosomal protein S25 | -0.2 | 0.00 |
| *Mrps30* | mitochondrial ribosomal protein S30 | -0.3 | 0.00 |
| *Mrps7* | mitochondrial ribosomal protein S7 | -0.2 | 0.04 |
| *Mterfd3* | MTERF domain containing 3 | -0.2 | 0.04 |
| *Mtfmt* | mitochondrial methionyl-tRNA formyltransferase | -0.5 | 0.00 |
| *Mtfp1* | mitochondrial fission process 1 | -0.3 | 0.03 |
| *Mtfr1* | mitochondrial fission regulator 1 | 0.7 | 0.00 |
| *Mtg1* | mitochondrial ribosome-associated GTPase 1 | -0.2 | 0.00 |
| *Mthfd2* | methylenetetrahydrofolate dehydrogenase (NADP+ dependent) 2, methenyltetrahydrofolate cyclohydrolase | 0.4 | 0.00 |
| *Mtif2* | mitochondrial translational initiation factor 2 | -0.2 | 0.05 |
| *Mtpap* | mitochondrial poly(A) polymerase | 0.2 | 0.03 |
| *Mtrf1l* | mitochondrial translational release factor 1-like | 0.7 | 0.00 |
| *Mtx2* | metaxin 2 | 0.4 | 0.05 |
| *Mutyh* | mutY homolog | 0.4 | 0.05 |
| *Myg1* | melanocyte proliferating gene 1 | -0.4 | 0.00 |
| *Nars* | asparaginyl-tRNA synthetase | 0.9 | 0.02 |
| *Ndor1* | NADPH dependent diflavin oxidoreductase 1 | -0.4 | 0.05 |
| *Ndufa1* | NADH dehydrogenase (ubiquinone) 1 alpha subcomplex, 1 | -0.2 | 0.00 |
| *Ndufa11* | NADH dehydrogenase (ubiquinone) 1 alpha subcomplex 11 | -0.2 | 0.00 |
| *Ndufa12* | NADH dehydrogenase (ubiquinone) 1 alpha subcomplex, 12 | -0.2 | 0.01 |
| *Ndufa13* | NADH dehydrogenase (ubiquinone) 1 alpha subcomplex, 13 | -0.3 | 0.01 |
| *Ndufa2* | NADH dehydrogenase (ubiquinone) 1 alpha subcomplex, 2 | -0.3 | 0.01 |
| *Ndufa3* | NADH dehydrogenase (ubiquinone) 1 alpha subcomplex, 3 | -0.6 | 0.00 |
| *Ndufa6* | NADH dehydrogenase (ubiquinone) 1 alpha subcomplex, 6 (B14) | -0.2 | 0.02 |
| *Ndufa8* | NADH dehydrogenase (ubiquinone) 1 alpha subcomplex, 8 | -0.4 | 0.00 |
| *Ndufaf2* | NADH dehydrogenase (ubiquinone) complex I, assembly factor 2 | 2.4 | 0.00 |
| *Ndufaf4* | NADH dehydrogenase (ubiquinone) complex I, assembly factor 4 | 1.3 | 0.03 |
| *Ndufaf5* | NADH dehydrogenase (ubiquinone) complex I, assembly factor 5 | -0.3 | 0.00 |
| *Ndufaf6* | NADH dehydrogenase (ubiquinone) complex I, assembly factor 6 | 0.3 | 0.04 |
| *Ndufaf7* | NADH dehydrogenase (ubiquinone) complex I, assembly factor 7 | 0.5 | 0.00 |
| *Ndufb10* | NADH dehydrogenase (ubiquinone) 1 beta subcomplex, 10 | -0.5 | 0.00 |
| *Ndufb3* | NADH dehydrogenase (ubiquinone) 1 beta subcomplex 3 | -0.2 | 0.04 |
| *Ndufb4* | NADH dehydrogenase (ubiquinone) 1 beta subcomplex 4 | -0.2 | 0.01 |
| *Ndufb7* | NADH dehydrogenase (ubiquinone) 1 beta subcomplex, 7 | -0.4 | 0.00 |
| *Ndufb8* | NADH dehydrogenase (ubiquinone) 1 beta subcomplex 8 | -0.3 | 0.00 |
| *Ndufc1* | NADH dehydrogenase (ubiquinone) 1, subcomplex unknown, 1 | 0.5 | 0.00 |
| *Ndufc2* | NADH dehydrogenase (ubiquinone) 1, subcomplex unknown, 2 | 0.4 | 0.00 |
| *Ndufs2* | NADH dehydrogenase (ubiquinone) Fe-S protein 2 | 0.6 | 0.00 |
| *Ndufs7* | NADH dehydrogenase (ubiquinone) Fe-S protein 7 | -0.5 | 0.00 |
| *Ndufv1* | NADH dehydrogenase (ubiquinone) flavoprotein 1 | -0.2 | 0.00 |
| *Ndufv3* | NADH dehydrogenase (ubiquinone) flavoprotein 3 | -0.3 | 0.01 |
| *Nfs1* | NFS1 nitrogen fixation 1 homolog (S. cerevisiae) | -0.4 | 0.00 |
| *Nif3l1* | NIF3 NGG1 interacting factor 3-like 1 (S. cerevisiae) | -0.6 | 0.00 |
| *Nit1* | nitrilase 1 | -0.3 | 0.05 |
| *Nit2* | nitrilase family, member 2 | -0.2 | 0.00 |
| *Nlrx1* | NLR family member X1 | -0.8 | 0.00 |
| *Nme3* | NME/NM23 nucleoside diphosphate kinase 3 | -0.4 | 0.00 |
| *Nme4* | non-metastatic cells 4, protein expressed in | -0.6 | 0.00 |
| *Nme6* | NME/NM23 nucleoside diphosphate kinase 6 | -0.7 | 0.00 |
| *Nrd1* | nardilysin 1 | -0.3 | 0.00 |
| *Nsun4* | NOP2/Sun domain family, member 4 | 0.8 | 0.02 |
| *Nt5dc2* | 5'-nucleotidase domain containing 2 | -0.5 | 0.00 |
| *Nthl1* | nth (endonuclease III)-like 1 (E.coli) | -0.6 | 0.00 |
| *Nudt13* | nudix (nucleoside diphosphate linked moiety X)-type motif 13 | -0.3 | 0.01 |
| *Nudt6* | nudix (nucleoside diphosphate linked moiety X)-type motif 6 | -0.6 | 0.01 |
| *Nudt8* | nudix (nucleoside diphosphate linked moiety X)-type motif 8 | -0.5 | 0.00 |
| *Nudt9* | nudix (nucleoside diphosphate linked moiety X)-type motif 9 | 1.1 | 0.00 |
| *Ogdh* | oxoglutarate (alpha-ketoglutarate) dehydrogenase (lipoamide) | 0.2 | 0.03 |
| *Ogg1* | 8-oxoguanine DNA glycosylase | -0.4 | 0.04 |
| *Opa1* | optic atrophy 1 | 0.3 | 0.04 |
| *Opa3* | optic atrophy 3 | 0.9 | 0.00 |
| *Oxa1l* | oxidase (cytochrome c) assembly 1-like | -0.4 | 0.01 |
| *Oxnad1* | oxidoreductase NAD-binding domain containing 1 | 5.5 | 0.00 |
| *Oxsm* | 3-oxoacyl-ACP synthase, mitochondrial | 0.6 | 0.00 |
| *Pak7* | p21 protein (Cdc42/Rac)-activated kinase 7 | 0.4 | 0.01 |
| *Pam16* | presequence translocase-associated motor 16 homolog (S. cerevisiae) | -0.4 | 0.00 |
| *Park2* | parkinson protein 2, E3 ubiquitin protein ligase | -0.2 | 0.02 |
| *Park7* | parkinson protein 7 | -0.3 | 0.00 |
| *Pars2* | prolyl-tRNA synthetase 2, mitochondrial (putative) | -0.3 | 0.01 |
| *Pccb* | propionyl CoA carboxylase, beta polypeptide | -0.3 | 0.02 |
| *Pck2* | phosphoenolpyruvate carboxykinase 2 (mitochondrial) | -0.5 | 0.00 |
| *Pdf* | peptide deformylase (mitochondrial) | -0.2 | 0.05 |
| *Pdha1* | pyruvate dehydrogenase (lipoamide) alpha 1 | 0.6 | 0.00 |
| *Pdhb* | pyruvate dehydrogenase (lipoamide) beta | 0.3 | 0.05 |
| *Pdhx* | pyruvate dehydrogenase complex, component X | -0.5 | 0.01 |
| *Pdk2* | pyruvate dehydrogenase kinase, isozyme 2 | -0.5 | 0.00 |
| *Pdp1* | pyruvate dehyrogenase phosphatase catalytic subunit 1 | 0.4 | 0.00 |
| *Pdp2* | pyruvate dehyrogenase phosphatase catalytic subunit 2 | 0.5 | 0.00 |
| *Pet100* | PET100 homolog (S. cerevisiae) | -0.4 | 0.00 |
| *Pgs1* | phosphatidylglycerophosphate synthase 1 | -0.4 | 0.01 |
| *Phb* | prohibitin | -0.2 | 0.00 |
| *Phyh* | phytanoyl-CoA 2-hydroxylase | 0.3 | 0.03 |
| *Pink1* | PTEN induced putative kinase 1 | -0.3 | 0.00 |
| *Plgrkt* | plasminogen receptor, C-terminal lysine transmembrane protein | -0.3 | 0.00 |
| *Pmaip1* | phorbol-12-myristate-13-acetate-induced protein 1 | 2.1 | 0.01 |
| *Pmpca* | peptidase (mitochondrial processing) alpha | -0.4 | 0.00 |
| *Polg* | polymerase (DNA directed), gamma | -0.2 | 0.01 |
| *Polg2* | polymerase (DNA directed), gamma 2, accessory subunit | -0.4 | 0.01 |
| *Polrmt* | polymerase (RNA) mitochondrial (DNA directed) | -0.6 | 0.00 |
| *Ppm1k* | protein phosphatase, Mg2+/Mn2+ dependent, 1K | 1.6 | 0.01 |
| *Ppm1m* | protein phosphatase, Mg2+/Mn2+ dependent, 1M | -0.5 | 0.00 |
| *Pptc7* | PTC7 protein phosphatase homolog (S. cerevisiae) | 0.6 | 0.01 |
| *Prelid1* | PRELI domain containing 1 | -0.3 | 0.00 |
| *Prodh2* | proline dehydrogenase (oxidase) 2 | -0.3 | 0.04 |
| *Prosc* | proline synthetase co-transcribed homolog (bacterial) | 0.2 | 0.00 |
| *Pstk* | phosphoseryl-tRNA kinase | -0.6 | 0.02 |
| *Ptcd1* | pentatricopeptide repeat domain 1 | -0.4 | 0.02 |
| *Ptcd2* | pentatricopeptide repeat domain 2 | 0.2 | 0.00 |
| *Ptpmt1* | protein tyrosine phosphatase, mitochondrial 1 | -0.2 | 0.00 |
| *Pus1* | pseudouridylate synthase 1 | -0.3 | 0.02 |
| *Pxmp2* | peroxisomal membrane protein 2 | -0.4 | 0.00 |
| *Pycr1* | pyrroline-5-carboxylate reductase 1 | -0.3 | 0.01 |
| *Pycr2* | pyrroline-5-carboxylate reductase family, member 2 | -0.3 | 0.03 |
| *Qrsl1* | glutaminyl-tRNA synthase (glutamine-hydrolyzing)-like 1 | -0.3 | 0.00 |
| *Qtrt1* | queuine tRNA-ribosyltransferase 1 | -0.5 | 0.00 |
| *Rab24* | RAB24, member RAS oncogene family | -0.4 | 0.00 |
| *Rab32* | RAB32, member RAS oncogene family | 1.2 | 0.01 |
| *Rars2* | arginyl-tRNA synthetase 2, mitochondrial | -0.2 | 0.00 |
| *Rfk* | riboflavin kinase | 0.8 | 0.03 |
| *Rhot2* | ras homolog family member T2 | -0.4 | 0.02 |
| *Rmnd1* | required for meiotic nuclear division 1 homolog (S. cerevisiae) | 0.4 | 0.00 |
| *Rnaseh1* | ribonuclease H1 | -0.4 | 0.00 |
| *Romo1* | reactive oxygen species modulator 1 | -0.4 | 0.00 |
| *Rpl10a* | ribosomal protein L10A | -0.2 | 0.00 |
| *Rpl34* | ribosomal protein L34 | -0.2 | 0.02 |
| *Rpl35a* | ribosomal protein L35a | -0.3 | 0.00 |
| *Rps14* | ribosomal protein S14 | -0.2 | 0.02 |
| *Rps15a* | ribosomal protein S15a | -0.3 | 0.01 |
| *Rps18* | ribosomal protein S18 | -0.3 | 0.00 |
| *Rpusd3* | RNA pseudouridylate synthase domain containing 3 | -0.4 | 0.00 |
| *Samm50* | SAMM50 sorting and assembly machinery component | -0.3 | 0.00 |
| *Sars2* | seryl-tRNA synthetase 2, mitochondrial | -0.4 | 0.00 |
| *Sco1* | SCO1 cytochrome c oxidase assembly protein | -0.4 | 0.00 |
| *Scp2* | sterol carrier protein 2 | -0.3 | 0.01 |
| *Sdhc* | succinate dehydrogenase complex, subunit C, integral membrane protein | 0.3 | 0.05 |
| *Sdr39u1* | short chain dehydrogenase/reductase family 39U, member 1 | -0.3 | 0.01 |
| *Secisbp2* | SECIS binding protein 2 | -0.4 | 0.04 |
| *Selo* | selenoprotein O | -0.3 | 0.00 |
| *Sept4* | septin 4 | -0.4 | 0.02 |
| *Sfxn4* | sideroflexin 4 | -0.5 | 0.01 |
| *Sirt3* | sirtuin 3 | -0.3 | 0.01 |
| *Sirt5* | sirtuin 5 | -0.2 | 0.04 |
| *Slc25a11* | solute carrier family 25 (mitochondrial carrier; oxoglutarate carrier), member 11 | -0.2 | 0.05 |
| *Slc25a12* | solute carrier family 25 (aspartate/glutamate carrier), member 12 | -0.2 | 0.01 |
| *Slc25a14* | solute carrier family 25 (mitochondrial carrier, brain), member 14 | -0.3 | 0.00 |
| *Slc25a16* | solute carrier family 25 (mitochondrial carrier, Graves disease autoantigen), member 16 | 0.3 | 0.01 |
| *Slc25a19* | solute carrier family 25 (mitochondrial thiamine pyrophosphate carrier), member 19 | -0.3 | 0.00 |
| *Slc25a20* | solute carrier family 25 (carnitine/acylcarnitine translocase), member 20 | -0.3 | 0.03 |
| *Slc25a23* | solute carrier family 25 (mitochondrial carrier; phosphate carrier), member 23 | -0.4 | 0.00 |
| *Slc25a24* | solute carrier family 25 (mitochondrial carrier, phosphate carrier), member 24 | 1.3 | 0.05 |
| *Slc25a26* | solute carrier family 25 (S-adenosylmethionine carrier), member 26 | -0.3 | 0.02 |
| *Slc25a28* | solute carrier family 25 (mitochondrial iron transporter), member 28 | -0.5 | 0.01 |
| *Slc25a29* | solute carrier family 25 (mitochondrial carnitine/acylcarnitine carrier), member 29 | -0.5 | 0.00 |
| *Slc25a3* | solute carrier family 25 (mitochondrial carrier, phosphate carrier), member 3 | 0.3 | 0.00 |
| *Slc25a30* | solute carrier family 25, member 30 | 0.5 | 0.02 |
| *Slc25a32* | solute carrier family 25 (mitochondrial folate carrier) , member 32 | 1.4 | 0.03 |
| *Slc25a33* | solute carrier family 25 (pyrimidine nucleotide carrier), member 33 | 0.6 | 0.00 |
| *Slc25a35* | solute carrier family 25, member 35 | -0.3 | 0.03 |
| *Slc25a37* | solute carrier family 25 (mitochondrial iron transporter), member 37 | 0.3 | 0.05 |
| *Slc25a39* | solute carrier family 25, member 39 | -0.5 | 0.00 |
| *Slc25a40* | solute carrier family 25, member 40 | -0.6 | 0.02 |
| *Slc25a42* | solute carrier family 25, member 42 | 0.6 | 0.00 |
| *Slc25a43* | solute carrier family 25, member 43 | 0.6 | 0.01 |
| *Slc25a44* | solute carrier family 25, member 44 | 0.3 | 0.00 |
| *Slc25a5* | solute carrier family 25 (mitochondrial carrier; adenine nucleotide translocator), member 5 | -0.2 | 0.05 |
| *Slc25a54* | solute carrier family 25, member 54 | -0.3 | 0.02 |
| *Slc30a6* | solute carrier family 30 (zinc transporter), member 6 | -0.5 | 0.00 |
| *Slc30a9* | solute carrier family 30 (zinc transporter), member 9 | 0.5 | 0.02 |
| *Slirp* | SRA stem-loop interacting RNA binding protein | -0.3 | 0.01 |
| *Smdt1* | single-pass membrane protein with aspartate-rich tail 1 | 0.5 | 0.00 |
| *Smim20* | small integral membrane protein 20 | -0.3 | 0.00 |
| *Snd1* | staphylococcal nuclease and tudor domain containing 1 | -0.3 | 0.01 |
| *Spata19* | spermatogenesis associated 19 | -1.0 | 0.00 |
| *Sqrdl* | sulfide quinone reductase-like (yeast) | -0.4 | 0.01 |
| *Stom* | stomatin | 0.9 | 0.02 |
| *Stoml2* | stomatin (Epb7.2)-like 2 | -0.2 | 0.02 |
| *Supv3l1* | suppressor of var1, 3-like 1 (S. cerevisiae) | -0.3 | 0.00 |
| *Surf1* | surfeit 1 | -0.2 | 0.01 |
| *Tars* | threonyl-tRNA synthetase | -0.3 | 0.00 |
| *Tars2* | threonyl-tRNA synthetase 2, mitochondrial (putative) | -0.3 | 0.02 |
| *Tcirg1* | T-cell, immune regulator 1, ATPase, H+ transporting, lysosomal V0 subunit A3 | -0.5 | 0.00 |
| *Tfam* | transcription factor A, mitochondrial | 0.3 | 0.00 |
| *Them4* | thioesterase superfamily member 4 | 0.7 | 0.00 |
| *Timm10b* | translocase of inner mitochondrial membrane 10 homolog B | -0.3 | 0.03 |
| *Timm13* | translocase of inner mitochondrial membrane 13 homolog (yeast) | -0.2 | 0.05 |
| *Timm17b* | translocase of inner mitochondrial membrane 17 homolog B (yeast) | -0.5 | 0.00 |
| *Timm23* | translocase of inner mitochondrial membrane 23 homolog (yeast) | 0.2 | 0.00 |
| *Timm8a1* | translocase of inner mitochondrial membrane 8 homolog A1 (yeast) | -0.3 | 0.00 |
| *Timm8a2* | translocase of inner mitochondrial membrane 8A2 | 1.2 | 0.00 |
| *Timm9* | translocase of inner mitochondrial membrane 9 homolog (yeast) | -0.3 | 0.00 |
| *Tk2* | thymidine kinase 2, mitochondrial | 0.5 | 0.04 |
| *Tmem126a* | transmembrane protein 126A | 0.6 | 0.04 |
| *Tmem143* | transmembrane protein 143 | -0.2 | 0.02 |
| *Tmem14c* | transmembrane protein 14C | -0.2 | 0.00 |
| *Tmem205* | transmembrane protein 205 | -0.2 | 0.01 |
| *Tmem223* | transmembrane protein 223 | -0.3 | 0.00 |
| *Tmem70* | transmembrane protein 70 | 0.3 | 0.04 |
| *Tomm6* | translocase of outer mitochondrial membrane 6 homolog (yeast) | -0.5 | 0.00 |
| *Tomm70a* | translocase of outer mitochondrial membrane 70 homolog A (S. cerevisiae) | 0.6 | 0.00 |
| *Trap1* | TNF receptor-associated protein 1 | -0.4 | 0.00 |
| *Trmt1* | tRNA methyltransferase 1 homolog (S. cerevisiae) | -0.5 | 0.02 |
| *Trmt10a* | tRNA methyltransferase 10 homolog A (S. cerevisiae) | 0.4 | 0.01 |
| *Trmt10c* | tRNA methyltransferase 10 homolog C (S. cerevisiae) | -0.3 | 0.01 |
| *Trub2* | TruB pseudouridine (psi) synthase family member 2 | -0.4 | 0.00 |
| *Tsfm* | Ts translation elongation factor, mitochondrial | 1.9 | 0.00 |
| *Tspo* | translocator protein | -0.6 | 0.01 |
| *Tst* | thiosulfate sulfurtransferase | -0.5 | 0.00 |
| *Ttc7b* | tetratricopeptide repeat domain 7B | -0.4 | 0.00 |
| *Tubb3* | tubulin, beta 3 class III | 0.4 | 0.01 |
| *Tufm* | Tu translation elongation factor, mitochondrial | -0.3 | 0.05 |
| *Txndc12* | thioredoxin domain containing 12 (endoplasmic reticulum) | -0.3 | 0.00 |
| *Txnrd2* | thioredoxin reductase 2 | -0.3 | 0.01 |
| *Tysnd1* | trypsin domain containing 1 | -0.5 | 0.00 |
| *Ucp1* | uncoupling protein 1 (mitochondrial, proton carrier) | -0.6 | 0.02 |
| *Ucp2* | uncoupling protein 2 (mitochondrial, proton carrier) | -0.8 | 0.02 |
| *Uqcc1* | ubiquinol-cytochrome c reductase complex assembly factor 1 | -0.4 | 0.00 |
| *Uqcc2* | ubiquinol-cytochrome c reductase complex assembly factor 2 | -0.3 | 0.00 |
| *Uqcr10* | ubiquinol-cytochrome c reductase, complex III subunit X | -0.2 | 0.00 |
| *Uqcr11* | ubiquinol-cytochrome c reductase, complex III subunit XI | -0.3 | 0.00 |
| *Uqcrc1* | ubiquinol-cytochrome c reductase core protein I | -0.2 | 0.01 |
| *Uqcrh* | ubiquinol-cytochrome c reductase hinge protein | -0.3 | 0.00 |
| *Uqcrq* | ubiquinol-cytochrome c reductase, complex III subunit VII | 1.0 | 0.00 |
| *Vdac3* | voltage-dependent anion channel 3 | 0.2 | 0.02 |
| *Vwa8* | von Willebrand factor A domain containing 8 | -0.3 | 0.04 |
| *Xpnpep3* | X-prolyl aminopeptidase (aminopeptidase P) 3, putative | -0.4 | 0.02 |
| *Yars2* | tyrosyl-tRNA synthetase 2 (mitochondrial) | 0.3 | 0.02 |
| *Yme1l1* | YME1-like 1 ATPase | 0.5 | 0.00 |
