# Supplementary material for: The Alterations in Mitochondrial DNA Copy Number and Nuclear-Encoded Mitochondrial Genes in Rat Brain Structures after Cocaine Self-Administration
Source: Mol Neurobiol. 2016 Nov 7;54(9):7460–70. doi: 10.1007/s12035-016-0153-3 (PMC5622911; doi:10.1007/s12035-016-0153-3)
Supplement: Supplementary file 3 — (DOCX 22 kb) [file 12035_2016_153_MOESM3_ESM.docx]

**Table S2. Nuclear genes encoding mitochondrial proteins with significant differential expression (FDR≤0.1; log_2_FC**≥**0.2 and ≤ -0.2) observed in rat hippocampus during the 3^rd^ day of extinction training after cocaine self-administration.**

| **Gene symbol** | **Gene name** | **log_2_ FC** | ***P* value** |
| --- | --- | --- | --- |
| *Abcb9* | ATP-binding cassette, subfamily B (MDR/TAP), member 9 | -0.6 | 0.00 |
| *Abcf2* | ATP-binding cassette, subfamily F (GCN20), member 2 | -0.3 | 0.00 |
| *Acad8* | acyl-CoA dehydrogenase family, member 8 | -0.4 | 0.02 |
| *Acadm* | acyl-CoA dehydrogenase, C-4 to C-12 straight chain | 0.2 | 0.00 |
| *Acadvl* | acyl-CoA dehydrogenase, very long chain | -0.2 | 0.01 |
| *Aco1* | aconitase 1, soluble | 0.2 | 0.01 |
| *Acot2* | acyl-CoA thioesterase 2 | 0.4 | 0.01 |
| *Acsm1* | acyl-CoA synthetase medium-chain family member 1 | 0.7 | 0.01 |
| *Acyp2* | acylphosphatase 2, muscle type | -0.2 | 0.02 |
| *Agk* | acylglycerol kinase | -0.3 | 0.02 |
| *Agpat5* | 1-acylglycerol-3-phosphate O-acyltransferase 5 | 0.5 | 0.00 |
| *Agxt2* | alanine-glyoxylate aminotransferase 2 | -1.0 | 0.01 |
| *Akr1b7* | aldo-keto reductase family 1, member B7 | 0.7 | 0.01 |
| *Aldh18a1* | aldehyde dehydrogenase 18 family, member A1 | 0.3 | 0.00 |
| *Aldh1l2* | aldehyde dehydrogenase 1 family, member L2 | 0.8 | 0.00 |
| *Aldh2* | aldehyde dehydrogenase 2 family (mitochondrial) | -0.4 | 0.02 |
| *Aldh7a1* | aldehyde dehydrogenase 7 family, member A1 | 0.3 | 0.00 |
| *Alkbh7* | alkB, alkylation repair homolog 7 (E. coli) | 0.5 | 0.01 |
| *Arl2* | ADP-ribosylation factor-like 2 | -0.6 | 0.01 |
| *Atp5l* | ATP synthase, H+ transporting, mitochondrial Fo complex, subunit G | 0.2 | 0.01 |
| *Atpaf2* | ATP synthase mitochondrial F1 complex assembly factor 2 | 0.2 | 0.02 |
| *Bax* | Bcl2-associated X protein | -0.3 | 0.01 |
| *Bcat1* | branched chain amino acid transaminase 1, cytosolic | -0.3 | 0.00 |
| *Bnip3l* | BCL2/adenovirus E1B interacting protein 3-like | 0.2 | 0.02 |
| *Bphl* | biphenyl hydrolase-like (serine hydrolase) | -0.2 | 0.02 |
| *Bphl* | biphenyl hydrolase-like (serine hydrolase) | -0.2 | 0.01 |
| *Car5b* | carbonic anhydrase 5b, mitochondrial | 0.4 | 0.01 |
| *Chchd1* | coiled-coil-helix-coiled-coil-helix domain containing 1 | 0.3 | 0.01 |
| *Chchd3* | coiled-coil-helix-coiled-coil-helix domain containing 3 | -0.5 | 0.01 |
| *Coq9* | coenzyme Q9 | 0.2 | 0.01 |
| *Cox4i2* | cytochrome c oxidase subunit IV isoform 2 (lung) | 0.3 | 0.02 |
| *Cox7b* | cytochrome c oxidase subunit VIIb | 0.2 | 0.01 |
| *Cox7c* | cytochrome c oxidase, subunit VIIc | 0.8 | 0.00 |
| *Cox8a* | cytochrome c oxidase subunit VIIIa | 0.2 | 0.00 |
| *Cox8a* | cytochrome c oxidase subunit VIIIa | 0.2 | 0.00 |
| *Ctu1* | cytosolic thiouridylase subunit 1 | -0.8 | 0.00 |
| *Cyb5b* | cytochrome b5 type B (outer mitochondrial membrane) | -0.4 | 0.00 |
| *Dap3* | death associated protein 3 | -0.4 | 0.00 |
| *Dars2* | aspartyl-tRNA synthetase 2 (mitochondrial) | 0.3 | 0.01 |
| *Dbi* | diazepam binding inhibitor (GABA receptor modulator, acyl-CoA binding protein) | -0.5 | 0.02 |
| *Decr1* | 2,4-dienoyl CoA reductase 1, mitochondrial | 0.3 | 0.01 |
| *Decr1* | 2,4-dienoyl CoA reductase 1, mitochondrial | 0.3 | 0.00 |
| *Diablo* | diablo, IAP-binding mitochondrial protein | 0.3 | 0.01 |
| *Dna2* | DNA replication helicase/nuclease 2 | 0.5 | 0.01 |
| *Dnajc11* | DnaJ (Hsp40) homolog, subfamily C, member 11 | -0.7 | 0.02 |
| *Dnajc4* | DnaJ (Hsp40) homolog, subfamily C, member 4 | -0.4 | 0.00 |
| *Ech1* | enoyl CoA hydratase 1, peroxisomal | -0.3 | 0.01 |
| *Eral1* | Era-like 12S mitochondrial rRNA chaperone 1 | -0.5 | 0.01 |
| *Fabp1* | fatty acid binding protein 1, liver | 1.0 | 0.01 |
| *Fbxl4* | F-box and leucine-rich repeat protein 4 | 0.3 | 0.00 |
| *Fdx1* | ferredoxin 1 | 0.2 | 0.01 |
| *Gk2* | glycerol kinase 2 | -1.1 | 0.01 |
| *Golph3* | golgi phosphoprotein 3 (coat-protein) | -0.2 | 0.01 |
| *Gpam* | glycerol-3-phosphate acyltransferase, mitochondrial | -0.8 | 0.02 |
| *Gpt2* | glutamic pyruvate transaminase (alanine aminotransferase) 2 | 1.2 | 0.00 |
| *Grpel2* | GrpE-like 2, mitochondrial | 0.5 | 0.01 |
| *Grpel2* | GrpE-like 2, mitochondrial | 0.3 | 0.01 |
| *Guk1* | guanylate kinase 1 | -0.4 | 0.01 |
| *Hdhd3* | haloacid dehalogenase-like hydrolase domain containing 3 | -0.4 | 0.01 |
| *Hk2* | hexokinase 2 | -1.0 | 0.00 |
| *Hsd3b1* | hydroxy-delta-5-steroid dehydrogenase, 3 beta- and steroid delta-isomerase 1 | -1.2 | 0.00 |
| *Hsdl1* | hydroxysteroid dehydrogenase like 1 | -1.6 | 0.02 |
| *Hsdl1* | hydroxysteroid dehydrogenase like 1 | -1.6 | 0.02 |
| *Hspb7* | heat shock protein family, member 7 (cardiovascular) | 1.3 | 0.00 |
| *Hspe1* | heat shock protein 1 (chaperonin 10) | 0.2 | 0.01 |
| *Isca2* | iron-sulfur cluster assembly 2 | -0.3 | 0.00 |
| *Lactb* | lactamase, beta | 0.8 | 0.00 |
| *Ldhal6b* | lactate dehydrogenase A-like 6B | 1.3 | 0.00 |
| *Letmd1* | LETM1 domain containing 1 | 0.2 | 0.00 |
| *Lyrm7* | LYR motif containing 7 | -0.6 | 0.01 |
| *Malsu1* | mitochondrial assembly of ribosomal large subunit 1 | 0.3 | 0.00 |
| *March5* | membrane-associated ring finger (C3HC4) 5 | -0.7 | 0.00 |
| *Mars2* | methionyl-tRNA synthetase 2, mitochondrial | 1.1 | 0.00 |
| *Mmab* | methylmalonic aciduria (cobalamin deficiency) cblB type | 1.4 | 0.00 |
| *Mrpl10* | mitochondrial ribosomal protein L10 | 0.4 | 0.00 |
| *Mrpl21* | mitochondrial ribosomal protein L21 | 0.4 | 0.00 |
| *Mrpl34* | mitochondrial ribosomal protein L34 | 0.3 | 0.01 |
| *Mrpl40* | mitochondrial ribosomal protein L40 | -0.2 | 0.01 |
| *Msra* | methionine sulfoxide reductase A | -0.6 | 0.02 |
| *Mthfd1* | methylenetetrahydrofolate dehydrogenase (NADP+ dependent) 1, methenyltetrahydrofolate cyclohydrolase, formyltetrahydrofolate synthetase | -0.2 | 0.02 |
| *Mtpap* | mitochondrial poly(A) polymerase | 0.4 | 0.01 |
| *Mtrf1l* | mitochondrial translational release factor 1-like | 0.4 | 0.00 |
| *Mul1* | mitochondrial E3 ubiquitin protein ligase 1 | -0.5 | 0.02 |
| *Ndufa10* | NADH dehydrogenase (ubiquinone) 1 alpha subcomplex 10 | 0.4 | 0.01 |
| *Ndufa8* | NADH dehydrogenase (ubiquinone) 1 alpha subcomplex, 8 | -0.3 | 0.01 |
| *Ndufaf2* | NADH dehydrogenase (ubiquinone) complex I, assembly factor 2 | 1.6 | 0.00 |
| *Ndufaf6* | NADH dehydrogenase (ubiquinone) complex I, assembly factor 6 | 0.4 | 0.00 |
| *Ndufaf7* | NADH dehydrogenase (ubiquinone) complex I, assembly factor 7 | 0.4 | 0.02 |
| *Ndufb3* | NADH dehydrogenase (ubiquinone) 1 beta subcomplex 3 | 0.3 | 0.02 |
| *Ndufb5* | NADH dehydrogenase (ubiquinone) 1 beta subcomplex, 5 | 0.2 | 0.00 |
| *Nme4* | non-metastatic cells 4, protein expressed in | 0.4 | 0.02 |
| *Nudt5* | nudix (nucleoside diphosphate linked moiety X)-type motif 5 | 0.3 | 0.01 |
| *Nudt9* | nudix (nucleoside diphosphate linked moiety X)-type motif 9 | 0.3 | 0.02 |
| *Ogdhl* | oxoglutarate dehydrogenase-like | -0.4 | 0.01 |
| *Oxct1* | 3-oxoacid CoA transferase 1 | 0.2 | 0.02 |
| *Oxnad1* | oxidoreductase NAD-binding domain containing 1 | 2.3 | 0.00 |
| *Pam16* | presequence translocase-associated motor 16 homolog (S. cerevisiae) | -0.5 | 0.01 |
| *Park2* | parkinson protein 2, E3 ubiquitin protein ligase | -0.7 | 0.00 |
| *Pdf* | peptide deformylase (mitochondrial) | 0.3 | 0.01 |
| *Pdhx* | pyruvate dehydrogenase complex, component X | 0.5 | 0.01 |
| *Pdp1* | pyruvate dehyrogenase phosphatase catalytic subunit 1 | 0.3 | 0.00 |
| *Phb* | prohibitin | 0.2 | 0.00 |
| *Pink1* | PTEN induced putative kinase 1 | -0.2 | 0.01 |
| *Pmpca* | peptidase (mitochondrial processing) alpha | -0.3 | 0.00 |
| *Polg* | polymerase (DNA directed), gamma | -0.4 | 0.01 |
| *Ptcd1* | pentatricopeptide repeat domain 1 | -0.7 | 0.02 |
| *Ptpmt1* | protein tyrosine phosphatase, mitochondrial 1 | -0.4 | 0.01 |
| *Ptpn4* | protein tyrosine phosphatase, non-receptor type 4 | -0.4 | 0.01 |
| *Pxmp4* | peroxisomal membrane protein 4 | 0.8 | 0.00 |
| *Pycr1* | pyrroline-5-carboxylate reductase 1 | 1.5 | 0.00 |
| *Rdh13* | retinol dehydrogenase 13 (all-trans/9-cis) | -0.7 | 0.00 |
| *Rps14* | ribosomal protein S14 | -0.3 | 0.01 |
| *Rpusd3* | RNA pseudouridylate synthase domain containing 3 | -0.6 | 0.01 |
| *Sdhc* | succinate dehydrogenase complex, subunit C, integral membrane protein | 0.2 | 0.01 |
| *Sfxn3* | sideroflexin 3 | 0.2 | 0.02 |
| *Sirt3* | sirtuin 3 | -0.5 | 0.01 |
| *Slc25a14* | solute carrier family 25 (mitochondrial carrier, brain), member 14 | -0.2 | 0.02 |
| *Slc25a16* | solute carrier family 25 (mitochondrial carrier, Graves disease autoantigen), member 16 | 0.3 | 0.00 |
| *Slc25a24* | solute carrier family 25 (mitochondrial carrier, phosphate carrier), member 24 | -1.4 | 0.00 |
| *Slc25a39* | solute carrier family 25, member 39 | -0.3 | 0.01 |
| *Slc25a44* | solute carrier family 25, member 44 | 0.4 | 0.00 |
| *Slmo2* | slowmo homolog 2 (Drosophila) | -0.3 | 0.00 |
| *Snd1* | staphylococcal nuclease and tudor domain containing 1 | -0.5 | 0.01 |
| *Sod2* | superoxide dismutase 2, mitochondrial | -0.5 | 0.02 |
| *Spata19* | spermatogenesis associated 19 | -0.7 | 0.01 |
| *Spata20* | spermatogenesis associated 20 | 1.2 | 0.00 |
| *Star* | steroidogenic acute regulatory protein | 0.7 | 0.01 |
| *Tfam* | transcription factor A, mitochondrial | 0.2 | 0.00 |
| *Timm13* | translocase of inner mitochondrial membrane 13 homolog (yeast) | -0.3 | 0.02 |
| *Tmem126a* | transmembrane protein 126A | 0.8 | 0.02 |
| *Tmem14c* | transmembrane protein 14C | -0.4 | 0.01 |
| *Tomm70a* | translocase of outer mitochondrial membrane 70 homolog A (S. cerevisiae) | -0.5 | 0.02 |
| *Tsfm* | Ts translation elongation factor, mitochondrial | 0.8 | 0.00 |
| *Ttc19* | tetratricopeptide repeat domain 19 | -0.7 | 0.01 |
| *Txnrd2* | thioredoxin reductase 2 | -0.4 | 0.02 |
| *Ucp1* | uncoupling protein 1 (mitochondrial, proton carrier) | -2.4 | 0.00 |
| *Uqcc1* | ubiquinol-cytochrome c reductase complex assembly factor 1 | -0.4 | 0.00 |
| *Uqcrb* | ubiquinol-cytochrome c reductase binding protein | 0.5 | 0.00 |
| *Uqcrq* | ubiquinol-cytochrome c reductase, complex III subunit VII | 1.4 | 0.00 |
| *Wars2* | tryptophanyl tRNA synthetase 2 (mitochondrial) | -1.0 | 0.00 |
