# Supplementary material for: The Alterations in Mitochondrial DNA Copy Number and Nuclear-Encoded Mitochondrial Genes in Rat Brain Structures after Cocaine Self-Administration
Source: Mol Neurobiol. 2016 Nov 7;54(9):7460–70. doi: 10.1007/s12035-016-0153-3 (PMC5622911; doi:10.1007/s12035-016-0153-3)
Supplement: Supplementary file 4 — (DOCX 19 kb) [file 12035_2016_153_MOESM4_ESM.docx]

**Table S3. Nuclear genes encoding mitochondrial proteins with significant differential expression (FDR≤0.1; log_2_FC**≥**0.2 and**

**≤ -0.2) observed in both rat brain structures during the 3^rd^ day of extinction training after cocaine self-administration.**

| **Gene symbol** | **Gene name** | **PFC** | **HP** |
| --- | --- | --- | --- |
|  |  |  |  |
| *Abcb9* | ATP-binding cassette, subfamily B (MDR/TAP), member 9 | ↓ | ↓ |
| *Acad8* | acyl-CoA dehydrogenase family, member 8 | ↓ | ↓ |
| ***Acadm*** | **acyl-CoA dehydrogenase, C-4 to C-12 straight chain** | **↓** | **↑** |
| *Acadvl* | acyl-CoA dehydrogenase, very long chain | ↓ | ↓ |
| *Acot2* | acyl-CoA thioesterase 2 | ↑ | ↑ |
| *Acyp2* | acylphosphatase 2, muscle type | ↓ | ↓ |
| *Agpat5* | 1-acylglycerol-3-phosphate O-acyltransferase 5 | ↑ | ↑ |
| *Agxt2* | alanine-glyoxylate aminotransferase 2 | ↓ | ↓ |
| ***Akr1b7*** | **aldo-keto reductase family 1, member B7** | **↓** | **↑** |
| *Aldh1l2* | aldehyde dehydrogenase 1 family, member L2 | ↑ | ↑ |
| *Aldh2* | aldehyde dehydrogenase 2 family (mitochondrial) | ↓ | ↓ |
| ***Aldh7a1*** | **aldehyde dehydrogenase 7 family, member A1** | **↓** | **↑** |
| ***Atp5l*** | **ATP synthase, H+ transporting, mitochondrial Fo complex, subunit G** | **↓** | **↑** |
| *Bax* | Bcl2-associated X protein | ↓ | ↓ |
| ***Bnip3l*** | **BCL2/adenovirus E1B interacting protein 3-like** | **↓** | **↑** |
| *Bphl* | biphenyl hydrolase-like (serine hydrolase) | ↓ | ↓ |
| *Car5b* | carbonic anhydrase 5b, mitochondrial | ↑ | ↑ |
| **Coq9** | **coenzyme Q9** | **↓** | **↑** |
| *Cox4i2* | cytochrome c oxidase subunit IV isoform 2 (lung) | ↑ | ↑ |
| *Cox7c* | cytochrome c oxidase, subunit VIIc | ↑ | ↑ |
| ***Cyb5b*** | **cytochrome b5 type B (outer mitochondrial membrane)** | **↑** | **↓** |
| *Dap3* | death associated protein 3 | ↓ | ↓ |
| *Dbi* | diazepam binding inhibitor (GABA receptor modulator, acyl-CoA binding protein) | ↓ | ↓ |
| ***Dna2*** | **DNA replication helicase/nuclease 2** | **↓** | **↑** |
| *Dnajc4* | DnaJ (Hsp40) homolog, subfamily C, member 4 | ↓ | ↓ |
| *Ech1* | enoyl CoA hydratase 1, peroxisomal | ↓ | ↓ |
| ***Golph3*** | **golgi phosphoprotein 3 (coat-protein)** | **↑** | **↓** |
| *Guk1* | guanylate kinase 1 | ↓ | ↓ |
| *Hdhd3* | haloacid dehalogenase-like hydrolase domain containing 3 | ↓ | ↓ |
| *Isca2* | iron-sulfur cluster assembly 2 | ↓ | ↓ |
| *Lactb* | lactamase, beta | ↑ | ↑ |
| ***Ldhal6b*** | **lactate dehydrogenase A-like 6B** | **↓** | **↑** |
| ***Letmd1*** | **LETM1 domain containing 1** | **↓** | **↑** |
| *Malsu1* | mitochondrial assembly of ribosomal large subunit 1 | ↑ | ↑ |
| *Mars2* | methionyl-tRNA synthetase 2, mitochondrial | ↑ | ↑ |
| *Mrpl10* | mitochondrial ribosomal protein L10 | ↑ | ↑ |
| *Mrpl21* | mitochondrial ribosomal protein L21 | ↑ | ↑ |
| *Mtpap* | mitochondrial poly(A) polymerase | ↑ | ↑ |
| *Mtrf1l* | mitochondrial translational release factor 1-like | ↑ | ↑ |
| *Ndufa8* | NADH dehydrogenase (ubiquinone) 1 alpha subcomplex, 8 | ↓ | ↓ |
| *Ndufaf2* | NADH dehydrogenase (ubiquinone) complex I, assembly factor 2 | ↑ | ↑ |
| *Ndufaf6* | NADH dehydrogenase (ubiquinone) complex I, assembly factor 6 | ↑ | ↑ |
| *Ndufaf7* | NADH dehydrogenase (ubiquinone) complex I, assembly factor 7 | ↑ | ↑ |
| ***Ndufb3*** | **NADH dehydrogenase (ubiquinone) 1 beta subcomplex 3** | **↓** | **↑** |
| ***Nme4*** | **non-metastatic cells 4, protein expressed in** | **↓** | **↑** |
| *Nudt9* | nudix (nucleoside diphosphate linked moiety X)-type motif 9 | ↑ | ↑ |
| *Oxnad1* | oxidoreductase NAD-binding domain containing 1 | ↑ | ↑ |
| *Pam16* | presequence translocase-associated motor 16 homolog (S. cerevisiae) | ↓ | ↓ |
| *Park2* | parkinson protein 2, E3 ubiquitin protein ligase | ↓ | ↓ |
| ***Pdf*** | **peptide deformylase (mitochondrial)** | **↓** | **↑** |
| ***Pdhx*** | **pyruvate dehydrogenase complex, component X** | **↓** | **↑** |
| *Pdp1* | pyruvate dehyrogenase phosphatase catalytic subunit 1 | ↑ | ↑ |
| ***Phb*** | **prohibitin** | **↓** | **↑** |
| *Pink1* | PTEN induced putative kinase 1 | ↓ | ↓ |
| *Pmpca* | peptidase (mitochondrial processing) alpha | ↓ | ↓ |
| *Polg* | polymerase (DNA directed), gamma | ↓ | ↓ |
| *Ptcd1* | pentatricopeptide repeat domain 1 | ↓ | ↓ |
| *Ptpmt1* | protein tyrosine phosphatase, mitochondrial 1 | ↓ | ↓ |
| ***Pycr1*** | **pyrroline-5-carboxylate reductase 1** | **↓** | **↑** |
| *Rps14* | ribosomal protein S14 | ↓ | ↓ |
| *Rpusd3* | RNA pseudouridylate synthase domain containing 3 | ↓ | ↓ |
| *Sdhc* | succinate dehydrogenase complex, subunit C, integral membrane protein | ↑ | ↑ |
| *Slc25a14* | solute carrier family 25 (mitochondrial carrier, brain), member 14 | ↓ | ↓ |
| *Slc25a16* | solute carrier family 25 (mitochondrial carrier, Graves disease autoantigen), member 16 | ↑ | ↑ |
| ***Slc25a24*** | **solute carrier family 25 (mitochondrial carrier, phosphate carrier), member 24** | **↑** | **↓** |
| Slc25a39 | solute carrier family 25, member 39 | ↓ | ↓ |
| *Slc25a44* | solute carrier family 25, member 44 | ↑ | ↑ |
| *Snd1* | staphylococcal nuclease and tudor domain containing 1 | ↓ | ↓ |
| *Spata19* | spermatogenesis associated 19 | ↓ | ↓ |
| *Tfam* | transcription factor A, mitochondrial | ↑ | ↑ |
| *Timm13* | translocase of inner mitochondrial membrane 13 homolog (yeast) | ↓ | ↓ |
| *Tmem126a* | transmembrane protein 126A | ↑ | ↑ |
| *Tmem14c* | transmembrane protein 14C | ↓ | ↓ |
| ***Tomm70a*** | **translocase of outer mitochondrial membrane 70 homolog A (S. cerevisiae)** | **↑** | **↓** |
| *Tsfm* | Ts translation elongation factor, mitochondrial | ↑ | ↑ |
| *Txnrd2* | thioredoxin reductase 2 | ↓ | ↓ |
| *Ucp1* | uncoupling protein 1 (mitochondrial, proton carrier) | ↓ | ↓ |
| *Uqcc1* | ubiquinol-cytochrome c reductase complex assembly factor 1 | ↓ | ↓ |
| *Uqcrq* | ubiquinol-cytochrome c reductase, complex III subunit VII | ↑ | ↑ |

In **bold** genes exhibiting different expression (up- or downregulation) in PFC and HP.
