# Supplementary material for: The Alterations in Mitochondrial DNA Copy Number and Nuclear-Encoded Mitochondrial Genes in Rat Brain Structures after Cocaine Self-Administration
Source: Mol Neurobiol. 2016 Nov 7;54(9):7460–70. doi: 10.1007/s12035-016-0153-3 (PMC5622911; doi:10.1007/s12035-016-0153-3)
Supplement: Supplementary file 5 — (DOCX 17 kb) [file 12035_2016_153_MOESM5_ESM.docx]

**Table S4. Nuclear-encoded OXPHOS related genes with significant differential expression (FDR≤0.1; log_2_FC≥0.2) observed in both rat brain structures during the 3^rd^ day of extinction training after cocaine self-administration.**

**PREFRONTAL CORTEX**

| **Gene symbol** | **Gene name** | **log_2_FC** |
| --- | --- | --- |
| **Respiratory chain complex I** | | |
| *Ndufa1* | NADH dehydrogenase (ubiquinone) 1 alpha subcomplex, 1 | -0.2 |
| *Ndufa11* | NADH dehydrogenase (ubiquinone) 1 alpha subcomplex 11 | -0.2 |
| *Ndufa12* | NADH dehydrogenase (ubiquinone) 1 alpha subcomplex, 12 | -0.2 |
| *Ndufa13* | NADH dehydrogenase (ubiquinone) 1 alpha subcomplex, 13 | -0.3 |
| *Ndufa2* | NADH dehydrogenase (ubiquinone) 1 alpha subcomplex, 2 | -0.3 |
| *Ndufa3* | NADH dehydrogenase (ubiquinone) 1 alpha subcomplex, 3 | -0.6 |
| *Ndufa6* | NADH dehydrogenase (ubiquinone) 1 alpha subcomplex, 6 (B14) | -0.2 |
| *Ndufa8* | NADH dehydrogenase (ubiquinone) 1 alpha subcomplex, 8 | -0.4 |
| *Ndufb10* | NADH dehydrogenase (ubiquinone) 1 beta subcomplex, 10 | -0.5 |
| *Ndufb3* | NADH dehydrogenase (ubiquinone) 1 beta subcomplex 3 | -0.2 |
| *Ndufb4* | NADH dehydrogenase (ubiquinone) 1 beta subcomplex 4 | -0.2 |
| *Ndufb7* | NADH dehydrogenase (ubiquinone) 1 beta subcomplex, 7 | -0.4 |
| *Ndufb8* | NADH dehydrogenase (ubiquinone) 1 beta subcomplex 8 | -0.3 |
| *Ndufc1* | NADH dehydrogenase (ubiquinone) 1, subcomplex unknown, 1 | 0.5 |
| *Ndufc2* | NADH dehydrogenase (ubiquinone) 1, subcomplex unknown, 2 | 0.4 |
| *Ndufs2* | NADH dehydrogenase (ubiquinone) Fe-S protein 2 | 0.6 |
| *Ndufs7* | NADH dehydrogenase (ubiquinone) Fe-S protein 7 | -0.5 |
| *Ndufv1* | NADH dehydrogenase (ubiquinone) flavoprotein 1 | -0.2 |
| *Ndufv3* | NADH dehydrogenase (ubiquinone) flavoprotein 3 | -0.3 |
| **Respiratory chain complex II** | | |
| *Sdhc* | succinate dehydrogenase complex, subunit C, integral membrane protein | 0.3 |
| **Respiratory chain complex III** | | |
| *Uqcr10* | ubiquinol-cytochrome c reductase, complex III subunit X | -0.2 |
| *Uqcr11* | ubiquinol-cytochrome c reductase, complex III subunit XI | -0.3 |
| *Uqcrc1* | ubiquinol-cytochrome c reductase core protein I | -0.2 |
| *Uqcrh* | ubiquinol-cytochrome c reductase hinge protein | -0.3 |
| *Uqcrq* | ubiquinol-cytochrome c reductase, complex III subunit VII | 1.0 |
| **Respiratory chain complex IV** | | |
| *Cox4i2* | cytochrome c oxidase subunit IV isoform 2 (lung) | 0.5 |
| *Cox5a* | cytochrome c oxidase, subunit Va | 0.4 |
| *Cox5b* | cytochrome c oxidase subunit Vb | -0.2 |
| *Cox6a2* | cytochrome c oxidase subunit VIa polypeptide 2 | -0.8 |
| *Cox6b2* | cytochrome c oxidase subunit VIb polypeptide 2 | 0.7 |
| *Cox6c* | cytochrome c oxidase, subunit VIc | -0.3 |
| *Cox7a2* | cytochrome c oxidase subunit VIIa polypeptide 2 | -0.4 |
| *Cox7c* | cytochrome c oxidase, subunit VIIc | 2.6 |
| *Cox8b* | cytochrome c oxidase, subunit VIIIb | -0.6 |
| **Respiratory chain complex V** | | |
| *Atp10d* | ATPase, class V, type 10D | -0.4 |
| *Atp5b* | ATP synthase, H+ transporting, mitochondrial F1 complex, beta polypeptide | 0.5 |
| *Atp5d* | ATP synthase, H+ transporting, mitochondrial F1 complex, delta subunit | -0.5 |
| *Atp5f1* | ATP synthase, H+ transporting, mitochondrial Fo complex, subunit B1 | 0.2 |
| *Atp5g2* | ATP synthase, H+ transporting, mitochondrial Fo complex, subunit C2 (subunit 9) | -0.2 |
| *Atp5g3* | ATP synthase, H+ transporting, mitochondrial Fo complex, subunit C3 (subunit 9) | 0.9 |
| *Atp5j* | ATP synthase, H+ transporting, mitochondrial Fo complex, subunit F6 | 0.2 |
| *Atp5j2* | ATP synthase, H+ transporting, mitochondrial Fo complex, subunit F2 | -0.3 |
| *Atp5l* | ATP synthase, H+ transporting, mitochondrial Fo complex, subunit G | -0.2 |
| **Coenzym Q** | | |
| *Coq10b* | coenzyme Q10 homolog B (S. cerevisiae) | 0.3 |
| *Coq6* | coenzyme Q6 monooxygenase | -0.4 |
| *Coq9* | coenzyme Q9 | -0.4 |
| **Respiratory chain assembly factors** | | |
| *Acad9* | acyl-CoA dehydrogenase family, member 9 | 0.2 |
| *Atpaf1* | ATP synthase mitochondrial F1 complex assembly factor 1 | 0.8 |
| *Coa3* | cytochrome C oxidase assembly factor 3 | 0.3 |
| *Coa4* | cytochrome c oxidase assembly factor 4 | -0.3 |
| *Cox11* | cytochrome c oxidase assembly homolog 11 (yeast) | 0.5 |
| *Cox14* | cytochrome c oxidase assembly protein 14 | -0.3 |
| *Cox16* | COX16 cytochrome c oxidase assembly homolog (S. cerevisiae) | 0.4 |
| *Cox17* | COX17 cytochrome c oxidase copper chaperone | -0.4 |
| *Cox18* | cytochrome c oxidase assembly protein 18 | -0.4 |
| *Cox19* | cytochrome c oxidase assembly homolog 19 (S. cerevisiae) | -0.2 |
| *Ecsit* | ECSIT signalling integrator | -0.4 |
| *Ndufaf2* | NADH dehydrogenase (ubiquinone) complex I, assembly factor 2 | 2.4 |
| *Ndufaf4* | NADH dehydrogenase (ubiquinone) complex I, assembly factor 4 | 1.3 |
| *Ndufaf5* | NADH dehydrogenase (ubiquinone) complex I, assembly factor 5 | -0.3 |
| *Ndufaf6* | NADH dehydrogenase (ubiquinone) complex I, assembly factor 6 | 0.3 |
| *Ndufaf7* | NADH dehydrogenase (ubiquinone) complex I, assembly factor 7 | 0.5 |
| *Sco1* | SCO1 cytochrome c oxidase assembly protein | -0.4 |
| *Surf1* | surfeit 1 | -0.2 |
| *Uqcc1* | ubiquinol-cytochrome c reductase complex assembly factor 1 | -0.4 |
| *Uqcc2* | ubiquinol-cytochrome c reductase complex assembly factor 2 | -0.3 |

**HIPPOCAMPUS**

| **Gene symbol** | **Gene name** | **log_2_FC** |
| --- | --- | --- |
| **Respiratory chain complex I** | | |
| *Ndufa10* | NADH dehydrogenase (ubiquinone) 1 alpha subcomplex 10 | 0.4 |
| *Ndufa8* | NADH dehydrogenase (ubiquinone) 1 alpha subcomplex, 8 | -0.3 |
| *Ndufb3* | NADH dehydrogenase (ubiquinone) 1 beta subcomplex 3 | 0.3 |
| *Ndufb5* | NADH dehydrogenase (ubiquinone) 1 beta subcomplex, 5 | 0.2 |
| **Respiratory chain complex I** | | |
| *Sdhc* | succinate dehydrogenase complex, subunit C, integral membrane protein | 0.2 |
| **Respiratory chain complex III** | | |
| *Uqcrb* | ubiquinol-cytochrome c reductase binding protein | 0.5 |
| *Uqcrq* | ubiquinol-cytochrome c reductase, complex III subunit VII | 1.4 |
| **Respiratory chain complex I** | | |
| *Cox4i2* | cytochrome c oxidase subunit IV isoform 2 (lung) | 0.3 |
| *Cox7b* | cytochrome c oxidase subunit VIIb | 0.2 |
| *Cox7c* | cytochrome c oxidase, subunit VIIc | 0.8 |
| *Cox8a* | cytochrome c oxidase subunit VIIIa | 0.2 |
| **Respiratory chain complex V** | | |
| *Atp5l* | ATP synthase, H+ transporting, mitochondrial Fo complex, subunit G | 0.2 |
| **Coenzym Q** | | |
| *Coq9* | coenzyme Q9 | 0.2 |
| **Respiratory chain assembly factors** | | |
| *Atpaf2* | ATP synthase mitochondrial F1 complex assembly factor 2 | 0.2 |
| *Ndufaf2* | NADH dehydrogenase (ubiquinone) complex I, assembly factor 2 | 1.6 |
| *Ndufaf6* | NADH dehydrogenase (ubiquinone) complex I, assembly factor 6 | 0.4 |
| *Ndufaf7* | NADH dehydrogenase (ubiquinone) complex I, assembly factor 7 | 0.4 |
| *Ttc19* | tetratricopeptide repeat domain 19 | -0.7 |
| *Uqcc1* | ubiquinol-cytochrome c reductase complex assembly factor 1 | -0.4 |
